# Efficacy and safety of insulin glargine 300 units/mL vs insulin degludec in patients with type 1 and type 2 diabetes: a systematic review and meta-analysis

**DOI:** 10.3389/fendo.2023.1285147

**Published:** 2024-01-19

**Authors:** Eman N. Alhmoud, Mohamed Omar Saad, Nabil Elhadi Omar

**Affiliations:** ^1^ Pharmacy Department, Al Wakra Hospital, Hamad Medical Corporation, Doha, Qatar; ^2^ Pharmacy Department, National Center for Cancer Care and Research, Hamad Medical Corporation, Doha, Qatar; ^3^ Health Sciences Program, Clinical and Population Health Research, College of Pharmacy, Qatar University, Doha, Qatar

**Keywords:** insulin, diabetes, glargine, degludec, hypoglycemia, HbA1c, fasting

## Abstract

**Background:**

Ultra-long-acting insulin analogs [insulin degludec (IDeg) and insulin glargine 300 units/mL (IGla-300)] offer a longer duration of action with less risk of hypoglycemia compared to other long-acting insulins. However, data about the comparative efficacy and safety are inconsistent.

**Methods:**

We searched CENTRAL, PubMed, Embase, ICTRP Search Portal, and ClinicalTrials.gov on 7 October 2022. Randomized controlled trials (RCTs) comparing the safety and efficacy of IDeg (100 or 200 units/mL) and IGla-300 in patients with type 1 or type 2 diabetes were included. Three review authors independently selected trials, assessed the risk of bias, extracted data, and evaluated the overall certainty of the evidence using GRADE. The primary outcomes were the change in glycated hemoglobin (HbA1c) and any hypoglycemia; the secondary outcomes were the change in fasting plasma glucose (FPG) and severe and nocturnal hypoglycemia.

**Results:**

Four open-label RCTs were included (2727 participants), 3 parallel and 1 cross-over. Overall, the risk of bias assessment yielded some concern or high risk. There was a comparable change in HbA1c from baseline to the end of treatment, a mean difference of 0.07% (95% confidence interval (CI) 0.06 – 0.19; p = 0.29; 3 trials; 2652 patients; very low-certainty evidence), and a comparable rate of any hypoglycemia, rate ratio 1.02 (95% CI 0.8 – 1.3; p = 0.87; 3 trials; 2881 patients; very low-certainty evidence). IDeg resulted in more reduction in FPG compared to IGla-300, mean difference of 10.27 mg/dL (95% CI 7.25 – 13.29; p < 0.001; 3 trials; 2668 patients; low-certainty evidence). Similar rates of nocturnal and severe hypoglycemia were observed, rate ratio of 1.13 (95% CI 0.72 – 1.78; p = 0.54; 3 trials; 2668 patients; very low-certainty evidence) and 1.4 (95% CI 0.41 – 4.73; p = 0.59; 2 trials; 1952 patients; very low-certainty evidence), respectively.

**Conclusion:**

There is no evidence of a difference between IDeg and IGla-300 in the mean change in HbA1c and the risk of anytime, nocturnal, and severe hypoglycemia. IDeg appeared to cause a higher reduction in FPG compared to IGla-300. However, this finding should be interpreted with caution due to the small number of trials included and their high risk of bias.

**Systematic review registration:**

https://www.crd.york.ac.uk/prospero/display_record.php?ID=CRD42022364891, identifier CRD42022364891.

## Background

### Description of the condition

More than 500 million people are living with diabetes. Furthermore, the number of people with diabetes is expected to reach 643 million by 2030 and 783 million by 2045. In 2021, 6.7 million deaths were attributed to diabetes (1). Diabetes is currently the greatest pandemic of the 21st century according to many epidemiologists (2, 3). The high global prevalence of diabetes has a vexing impact on individuals, healthcare systems, and countries all over the globe (4). Not only can diabetes decrease patients’ quality of life, it can also increase the chance of premature death (1).

### Description of the intervention

Insulin therapy is the mainstay of the management of type 1 diabetes and intensive glucose control in individuals with type 1 diabetes (glycated hemoglobin (HbA1c) < 7%) has been proven to decrease microvascular and macrovascular complications (5–8). Insulin replacement regimens in type 1 diabetes typically consist of basal insulin, mealtime insulin, and correction insulin. Basal insulin options include NPH intermediate-acting insulin, long-acting insulin analogs, and the continuous delivery of rapid-acting insulin via an insulin pump (9).

Moreover, due to the progressive nature of type 2 diabetes, the need for therapy intensification through the addition of insulin is common, particularly with longer disease duration. In type 2 diabetes, basal insulin is typically added to background therapy of oral and/or injectable glucose-lowering agents, with subsequent therapy intensification decided by the degree of blood glucose control.

The second-generation basal insulin (BI) analogs, insulin degludec (IDeg), and concentrated insulin glargine-300 units/mL (IGla-300) represented a significant revolution in the management of patients with diabetes (10). Compared to first-generation basal insulin glargine-100 units/mL (IGla-100), both insulins display flatter, more constant, and prolonged action in a steady state (11, 12). These properties are explained by their advanced modes of protraction.

IDeg is the only insulin analog that self-associates into multi-hexamers upon subcutaneous injection. This results in soluble depot formation from which IDeg monomers are slowly and steadily dissociated and released into the circulation. IDeg is available in two bioequivalent strengths: 100 units/mL and 200 U/mL. Switching from one to another does not require adjustment in the dose (13).

IGla-300, on the other hand, delivers the same number of insulin units as IGla-100, but in one-third of the injection volume. Following subcutaneous administration, IGla-300 forms a smaller precipitate than IGla-100. The smaller surface area of IGla-300 leads to slower and prolonged release of insulin from the subcutaneous depot at the injection site (10). IGla-300 is not bioequivalent to IGla-100, and dose adjustment is required upon switching between the two agents or other basal insulins (14).

### Importance of the current review

Compared to IGla-100, both IDeg and IGla-300 achieve comparable glycemic control with lower rates of confirmed and severe hypoglycemia (anytime and nocturnal) in patients with type 2 diabetes mellitus (15–17). Glycemic control was even more sustained at 1 year with Gla-300 (16).

In patients with type 1 diabetes, IGla300 was associated with significantly lower nocturnal and severe hypoglycemia along with comparable or slightly better reductions in HbA1c (18, 19).

In contrast, a recent Cochrane review found no evidence of difference in severe hypoglycemia and severe nocturnal hypoglycemia between IDeg and IGla-100 in patients with type 1 diabetes, with a higher reduction in HbA1c observed with IGla-100 compared to IDeg (20).

Head-to-head comparisons between IDeg and IGla-300 are limited. A recent systematic review and meta-analysis by Yang et al. compared IDeg and insulin glargine (both 100 and 300 units/mL) in terms of glycemic variability among patients with type 1 and type 2 diabetes (21). Overall, this review demonstrated insignificant difference between insulin glargine and IDeg in 24-hour mean and standard deviation of blood glucose, mean amplitude of glycemic excursion (MAGE), coefficient of Variation (CV) of 24-h blood glucose, mean of daily differences (MODD), and area under the glucose curve. Time in the therapeutic range in IDeg 100 was longer than Igla-100 but not Igla-300. Moreover, mean fasting plasma glucose (FPG) was lower with Ideg compared to insulin glargine. Nonetheless, the lack of identification of insulin glargine formulation was a major limitation of this review. Only 5 out of the 14 included studies in the review by Yang et al. evaluated Igla-300 specifically, whereas the type of insulin glargine was not identified in 6 studies (21).

Considering the differences in PK/PD parameters and clinical outcomes of Igla-100 and Igla-300, pooling the two insulins and treating them as a single molecule is questionable (11, 12). Additionally, Yang et al. included mostly small-size studies conducted in China and Japan, which limits the external generalizability of the findings. Moreover, the evaluated outcomes depended vastly on data obtained from continuous glucose monitoring (CGM) devices or several daily tests via self-monitoring of blood glucose (SMBG), which may not be readily available or feasible in routine care. Furthermore, the review did not evaluate the incidence and/or rate of hypoglycemia, a clinically important, patient-centered outcome (21).

A more recent systematic review and meta-analysis by Dong et al. that compared Ideg as an intervention against other long-acting basal insulin analogs (insulin glargine (Igla-100 and Igla-300) and insulin detemir, collectively) revealed overall comparable reduction in HbA1c and severe hypoglycemia between the two groups (22). FPG, overall, and nocturnal hypoglycemia, were significantly lower with Ideg in the combined group of type 1 and type 2 diabetes patients (22). However, the review by Dong et al. had many limitations. Firstly, there was a lack of identification of insulin glargine formulation. Secondly, no studies of Igla-300 in type 1 diabetes were evaluated. Additionally, severe hypoglycemia was defined by a cutoff blood glucose value of <2.9 mmol/L, which contradicts the internationally accepted definition of altered mental and/or physical functioning that necessitates assistance from another person for recovery (23). The search strategy was not comprehensively described and appeared to be limited to four databases, without any consideration of gray, unpublished literature. Furthermore, the decision between fixed and random effects analysis was based on statistical testing for heterogeneity, which is strongly discouraged by the Cochrane group (22, 24).

Moreover, cost-effectiveness comparisons between Ideg and Igla-300 in patients with type 1 and type 2 diabetes provided contradicting results (25–27).

Thus, there is currently a lack of scientific literature specifically comparing Ideg and Igla-300. Understanding the comparative efficacy and safety of these two agents is vitally important to patients, clinicians, and decision-makers.

## Objectives

To compare the effects of Igla-300 to Ideg (100 or 200 units/mL) on glycemic control (reduction in HbA1c and FPG) and risk of hypoglycemia (anytime, severe, and nocturnal hypoglycemia) in patients with type 1 and type 2 diabetes mellitus.

## Methods

### Criteria for considering studies for this review

#### Types of studies

We included randomized controlled trials (RCTs). Observational studies, case reports, case series, pharmacokinetics studies, pharmacodynamic studies, and animal studies were excluded.

#### Types of participants (population)

We included trials of adult and pediatric patients diagnosed with type 1 diabetes or type 2 diabetes. Both insulin-naïve patients and patients switching from other basal insulins were included.

We excluded hospitalized patients as findings in hospitalized patients may not be generalizable to others due to fluctuating plasma glucose, disrupted eating patterns, stress accompanying acute illnesses, and uncertainty around goal glycemic targets.

#### Types of interventions

##### Intervention

Insulin degludec (Ideg) 100 or 200 units/mL, administered by subcutaneous injection once daily.

##### Comparator

Insulin glargine (Igla-300) 300 units/mL, administered by subcutaneous injection once daily.

We excluded trials evaluating formulations of combined insulin and glucagon-like peptide 1 (GLP-1) receptor agonists and/or rapid-acting insulins as findings in patients receiving combination products may not be generalizable to others.

#### Types of outcome measures/Method of outcome measurement/Timing of outcome measurement

We included trials with defined clinical outcomes related to glycemic control and hypoglycemia.

##### The primary outcomes

Change in HbA1c (reported as a percentage or mmol/mol) over a minimum treatment duration of 3 months

Anytime hypoglycemia events [defined as blood glucose (BG) less than 70 mg/dl (3.9 mmol/L)]

##### Secondary outcomes

Change in FPG at a minimum of 3-months duration.

Severe hypoglycemia events as defined in the included studies, throughout the treatment period.

Nocturnal hypoglycemia events, defined as hypoglycemia during the night, throughout the treatment period, as reported in studies.

Randomized controlled trials reporting solely pharmacodynamic and/or pharmacokinetic outcomes (e.g., steady state insulin concentration and glucose infusion rate profiles) and studies evaluating measures of glycemic variability only (e.g., standard deviation, coefficient of variation, the mean time within the target glucose range, and the mean percentage of time with hypoglycemia or hyperglycemia) were excluded.

### Search methods for identification of studies

#### Electronic searches (information sources)

Three reviewers searched the following sources using a time frame starting from the inception of each database to the date of search. We did not place restrictions on the language of publication:

- Cochrane Central Register of Controlled Trials (CENTRAL) via the Cochrane Register of Studies Online (CRSO) (searched 07 October 2022)
- PubMed database (searched 07 October 2022)
- Embase biomedical research (Elsevier) (searched 07 October 2022)
- ClinicalTrials.gov (www.clinicaltrials.gov) (searched 07 October 2022)
- World Health Organization International Clinical Trials Registry Platform (ICTRP) (www.who.int/trialsearch) (searched 07 October 2022)

We searched gray literature using:

- OpenGrey (https://opengrey.eu/) (searched 07 October 2022)
- Web of Conferences (https://www.webofconferences.org/) (searched 07 October 2022)
- Google Scholar (https://scholar.google.com) (searched 07 October 2022)
- ProQuest Dissertations and Thesis Global (https://www.proquest.com/) (searched 07 October 2022)
- Study synopses provided by the drug manufacturer websites (e.g., https://www.novonordisk-trials.com/)

We identified other potentially eligible studies by searching the reference lists of included studies, systematic reviews, and meta-analyses retrieved for full article review. Additionally, if a published protocol of a potentially relevant study was marked as a completed trial in a clinical trials registry such as ClinicalTrials.gov, we contacted the primary author to enquire about publication status and request unpublished data, if available.

Finally, we updated the database search on 30^th^ December 2022 using the same search method, for the dates after 7^th^ October 2022. Details of search strategies are listed in **Appendix 1**.

### Selection process

Search results from databases and registries were combined on Endnote, where duplicate records were removed; then unique records were exported to Rayyan (28) (www.rayyan.ai). On Rayyan, three reviewers independently screened the titles and abstracts of the records and assessed eligibility for inclusion in the review. Each reviewer was blinded to the decisions of other reviewers. Three reviewers independently reviewed the full text of relevant reports identified through title/abstract screening. We excluded records if they had publication type, study design, population, interventions, or outcomes that did not match the previously mentioned criteria. We also excluded review articles, background articles, and exact duplicate records. However, systematic review/meta-analysis articles were identified to be screened for relevant primary reports in their reference lists. Disagreement between reviewers was resolved by discussion and consensus.

### Data collection and analysis

The process of data collection and analysis was conducted in accordance with Cochrane guidelines and in different steps; these steps included data extraction, assessment of risk of bias in included studies, measurement of treatment effects, dealing with missing data, data synthesis, assessment of heterogeneity, subgroup analysis and investigation of heterogeneity, assessment of reporting bias, and assessment of the certainty of evidence.

The process of data collection and analysis is summarized in **Appendix 2** and is detailed below.

#### Data extraction

For studies that fulfilled our inclusion criteria, three reviewers extracted the relevant data independently, and each reviewer’s extracted data were double-checked by another reviewer for accuracy. Disagreements on the extracted data were resolved through discussion and consensus. Data extraction was done using a specifically designed data extraction form on MS Excel. We planned to contact the study authors for any missing information. We extracted the following data from the included studies:

1. Study characteristics (last name of the first author, publication year, ClinicalTrials.gov or WHO ICTRP identifier code, follow-up duration, sample size in each group and the details of interventions and comparisons, primary and secondary outcomes).
2. Patient characteristics (key inclusion and exclusion criteria, baseline HbA1c, baseline duration of diabetes, previous treatments, average age, sex, and other key features).
3. Interventions: we described interventions according to an adapted version of the ‘template for intervention description and replication’ (TIDieR) checklist (29, 30), which was reported in a Cochrane review by Hemmingsen B, Metzendorf MI, Richter B (20).
4. Outcomes of interest: details are provided in ‘Measures of treatment effects’
5. Funding sources

#### Data from clinical trials registers

We extracted data for the included studies if they were available as study results in clinical trials registers, such as ClinicalTrials.gov, and not reported in the published report.

#### Assessment of risk of bias in included studies

Three reviewers assessed the quality of included studies and evaluated the risk of bias, independently, using version 2 of the Cochrane risk-of-bias tool for randomized trials (RoB 2) (31, 32). The tool is domain-based and consists of five domains with a set of signaling questions that guide reviewers in assessing the risk of bias in the following domains:

- Domain 1: bias arising from the randomization process.
- Domain 2: bias due to deviations from the intended interventions (effect of assignment or adherence to intervention).
- Domain 3: bias due to missing outcome data.
- Domain 4: bias in measurement of the outcome.
- Domain 5: bias from selection of reported results

For the cross-over trial, the appropriate RoB 2 tool was used, which has an additional domain for bias arising from the washout period and carryover effects. We evaluated the risk of bias in the effect of assignment to the interventions at baseline (intention-to-treat effect).

Based on the domain’s ratings, the risk of bias in randomized trials was rated as ‘low’, ‘high’, or ‘some concern’. The risk of bias judgment was established for each specific outcome using the criteria set in the RoB 2 tool. Reasons that support the reviewers’ judgment were also reported. Disagreements were solved by discussion and consensus between the reviewers. We emailed the corresponding author of one trial (BRIGHT) to seek clarification about differences between intended and reported methodologies and outcomes, but we did not get a response (33).

#### Measures of treatment effects

For continuous outcomes (change in HbA1c, change in FPG), we extracted the means (or least square means) with their corresponding standard deviation (SD) for each group. We used the standard error and the number of subjects analyzed to calculate SD when it was not reported. We used the reported mean differences (or least square mean differences) and their corresponding 95% CIs to calculate the pooled effect estimate. One trial (CONCLUDE) (34) used IGla-300 as the reference group, so we converted its reported mean difference and its corresponding 95% CI by subtracting from zero to calculate the effect estimate of IGla-300 vs. IDeg.

For hypoglycemic event rates expressed as events per patient-year, we extracted the number of events and the rate of hypoglycemia for each group. We converted all hypoglycemia event rates to (events per patient-year) by multiplying the (events per patient-week) by 52 and dividing (events per 100 patient-years) by 100. We used the reported rate ratios and their corresponding 95% CIs) to calculate the pooled effect estimate. One trial (CONCLUDE) (34) used IGla-300 as the reference group, so we converted its reported rate ratio and its corresponding 95% CI by calculating the reciprocal to calculate the effect estimate of Igla-300 vs. Ideg.

#### Dealing with missing data

We contacted the primary authors to obtain missing data. Important numerical data such as number of subjects screened, number randomly assigned, and intention-to-treat (ITT), as-treated or per-protocol populations were thoroughly evaluated. We critically appraised issues related to missing data as part of the risk of bias assessment. We did not do imputations for missing data on study subjects.

#### Data synthesis

Outcomes in individual groups of studies, treatment effect estimates of individual studies, and pooled effect estimates were presented in forest plots separately for each outcome.

Treatment effect estimates were pooled by random-effects model to account for between-study variations due to differences in type and duration of diabetes, concomitant treatments, age, and comorbidities of participants. Between-studies variation (Tau^2^) was estimated by the restricted maximum likelihood approach (REML). A prediction interval, which requires at least three studies, was synthesized to specify a predicted range for the true treatment effect in future individual studies (35). It is noteworthy that the Cochrane group recommends a “reasonable number of about 10 studies” to estimate prediction interval (24). We did not perform any sensitivity analyses.

We performed statistical analyses according to the statistical guidelines presented in the Cochrane Handbook for Systematic Reviews of Interventions (24). All statistical analyses were conducted using Stata version 17.

#### Assessment of heterogeneity

Heterogeneity was assessed by visual inspection of the forest plot and by using the standard χ² test (with a significance level of α of 0.1) (24).

Chi^2^ tests must be interpreted with caution due to low power in the situation of a meta-analysis when studies have a small sample size or are few. Thus, quantification of heterogeneity was estimated by I^2^ statistic to assess the impact of heterogeneity on the meta-analysis (24).

#### Subgroup analysis and investigation of heterogeneity

In case of substantial heterogeneity, we examined Galbraith plots to identify outlier studies. Due to differences in the etiology, age at onset, concomitant medications, and the potential impact of residual insulin activity; a subgroup meta-analysis for type-1 and type-2 diabetes separately was decided *a priori* to assess the effect of the type of diabetes on the pooled effect measures. However, due to the small number of included studies, subgroup analysis was not conducted. Meta-regression was not considered as the number of studies in the meta-analysis was fewer than 10.

#### Assessment of reporting biases

We planned to assess reporting bias through visual assessment of a funnel plot asymmetry with careful consideration of other factors that could explain funnel plot asymmetry, such as heterogeneity and poor methodological design (small study bias) (36).

Contour-enhanced funnel plot was also planned to differentiate asymmetry that is due to non-reporting biases from that due to other factors.

A formal assessment of publication bias could not be conducted by Egger’s test as the required criteria for the test were not met (24) (minimum of 10 studies). The trim-and-fill method was planned to impute missing effect estimates due to publication bias, but it did not yield any additional estimates.

#### Assessment of certainty of evidence

We assessed the certainty of the evidence for each outcome specified below, according to the Grading of Recommendations Assessments, Development, and Evaluation (GRADE) tool (37). Two review authors (EA, MS) independently rated the certainty of the evidence for each outcome. Disagreements on ratings were resolved through discussion and consensus. A summary of the GRADE evidence profile was reported.

## Results

### Results of the search

Studies that reported prespecified primary and/or secondary outcomes of efficacy (change in HbA1c and FPG) and safety (anytime, nocturnal, and severe hypoglycemia) were eligible for data synthesis.

The initial database search identified 1453 records. Excluding studies that did not meet the inclusion criteria through title/abstract search yielded 49 records that were sought for retrieval. Of which, we contacted the primary authors of 7 records but did not get a reply (one published abstract, three completed/terminated studies: JPRN-UMIN0000199693, UMIN000025952, UMIN000025122, and three ongoing studies: UMIN000019525, EudraCT number 2016-002725-11, UMIN000026829). A search of other sources (gray literature) identified 236 records.

Full-text screening from the electronic search and additional sources yielded 22 reports of four eligible randomized clinical trials (RCTs). The update search on January 3^rd^, 2023, prior to publication yielded (Cochrane=19, PubMed=7, and Embase=15 records), of which none were considered eligible.

**Figure 1:**
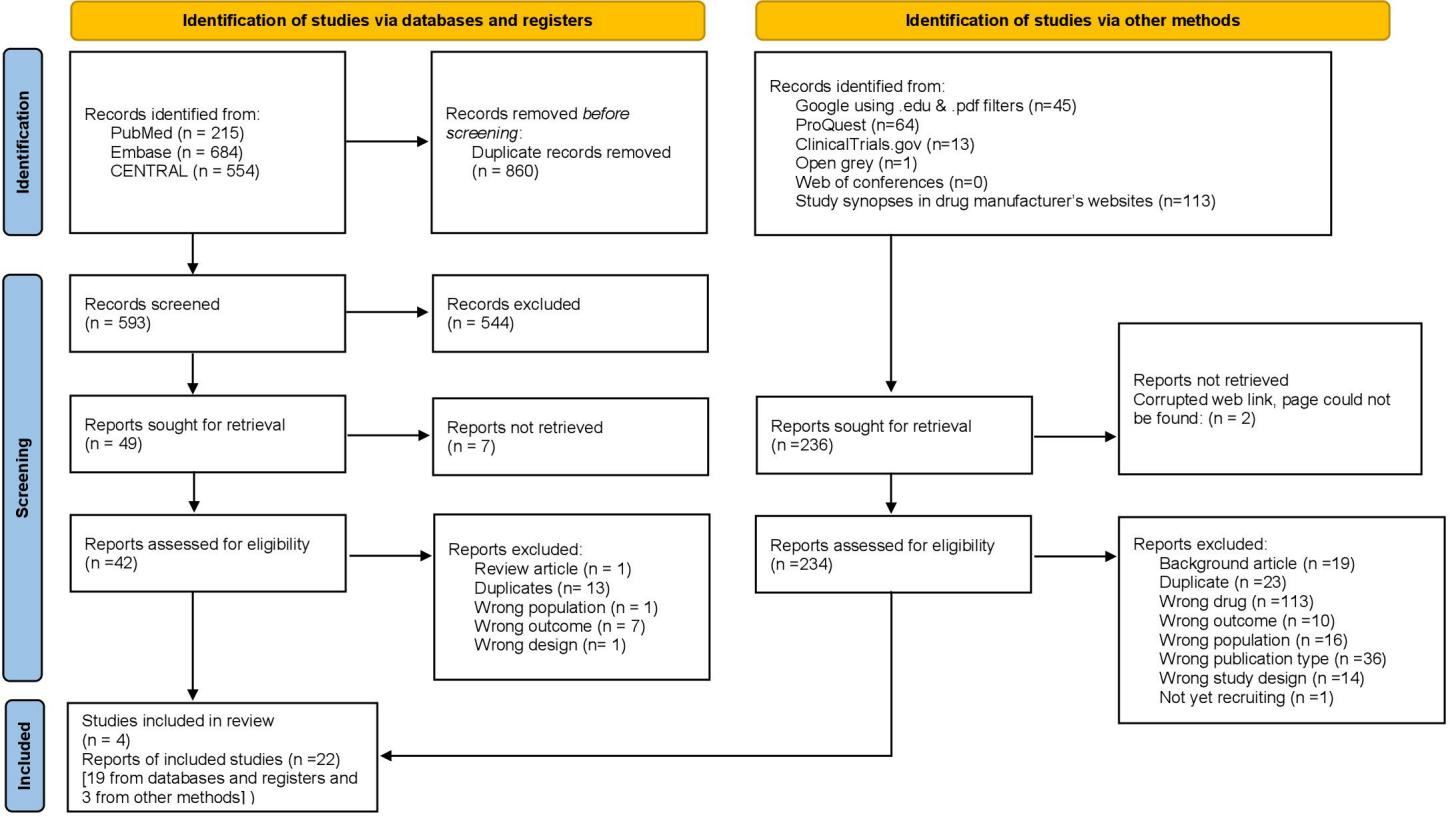
PRISMA flow diagram showing the process of study selection and the reasons for exclusion.

The process of study selection and the reasons for record exclusion are illustrated by the updated PRISMA flow diagram (**Figure 1**) (38).

### Overview of study populations

The details of the included trials are described in **Appendix 3**.

A total of 2727 participants were randomized in four trials: 1465 participants to Igla-300 and 1462 participants to Ideg. All trials evaluated adults, two studies included participants with type 1 diabetes, and two studies included participants with type 2 diabetes.

### Study designs

Three studies were parallel, head-to-head RCTs (BRIGHT, CONCLUDE, and InRange trials) (33, 34, 39) and one study had a cross-over design (Kobe Best Basal Insulin Study 2) (40). All studies implemented an open-label design. The duration of the intervention ranged from 4 weeks to 88 weeks. All studies were multinational, except one (Kobe Best Basal Insulin Study 2) (40). All studies were multicenter. The number of study centers ranged from 14 (Kobe Best Basal Insulin Study 2) (40) to 229 (CONCLUDE trial) (34). None of the studies terminated early. One trial (CONCLUDE) (34) underwent a protocol amendment due to the discovered inaccuracy of glycemic data collection systems, which were replaced by conventional glucometers. This necessitated an extension of the study duration for an additional 36-week maintenance period.

### Participants

All studies included both sex. The duration of diabetes ranged from 10.5 to 20.7 years. Descriptions of baseline characteristics of the participants are presented in **Table 1**.

**Table 1:** Baseline characteristics of study subjects (studies ordered according to year of publication).

Study ID	Groups	No. of subjects	Age (years) Mean (SD)	Sex Male(%)	HbA1c% Mean (SD)	BMI (Kg/m2) Mean (SD)	Diabetes duration (years) Mean (SD)
Rosenstock J, 2018	Igla300	466	60.6 (9.6)	53	8.71 (0.83)	31.7 (4.3)	10.5 (6.1)
	Ideg	463	60.5 (9.8)	54	8.57 (0.80)	31.3 (4.4)	10.7 (6.5)
Philis-Tsimikas A, 2020	Igla300	804	62.8 (10.0)	54.2	7.6 (0.9)	31.5 (5.2)	15.1 (8.2)
	Ideg	805	62.9 (10.0)	58.6	7.6 (1.0)	31.7 (5.3)	15.0 (8.4)
Miura, 2020*	Igla300/Ideg	46	53.3 (14.7)	30.4	7.6 (0.7)	22.2 (3.4)	19.4 (11.6)
	Ideg/Igla300						
Battelino T, 2022	Igla300	172	42.9 (13.53)	50	8.29 (0.82)	27.6 (5.07)	20.7 (12.47)
	Ideg	171	42.8 (13.05)	56.7	8.34 (0.80)	27.0 (4.44)	20.3 (13.12)

*Baseline characteristics reported for whole study participants.

#### Studies of participants with type 2 diabetes

Studies of participants with type 2 diabetes (n=2) enrolled insulin naïve patients previously uncontrolled with oral hypoglycemic agents (OHAs) +/- glucagon-like peptide 1 receptor agonists (GLP1RA) (BRIGHT trial) (33) or patients previously treated with basal insulin +/- OHAs (Philis-Tsimikas) (34). Participants enrolled in the two trials of type 2 diabetes differed in their baseline risk of hypoglycemia. The BRIGHT trial (33) excluded patients with a history of hypoglycemia unawareness or repeated episodes of severe hypoglycemia, whereas subjects in the CONCLUDE trial had to fulfill at least one of the following criteria of increased hypoglycemia risk: severe hypoglycemia within the preceding year, moderate chronic renal failure (glomerular filtration rate 30-59 mL/min/1.73m^2^), unawareness of hypoglycemic symptoms, treatment with insulin for >5 years, or a hypoglycemia episode within the last 12 weeks prior to the screening visit. Participants in the BRIGHT trial had a higher baseline HbA1c and a shorter duration of diabetes (33).

#### Studies of participants with type 1 diabetes:

The remaining two studies (39, 40) included patients with type 1 diabetes previously treated with basal/bolus insulin regimens. Patients varied in their baseline HbA1c (8.3% vs. 7.6%) and body mass index (BMI) (22.2 Kg/m^2^ vs ~ 27 Kg/m^2^).

Overall, participants in type 1 diabetes trials were younger than those in type 2 diabetes (age range 42.8 to 53.3 years versus 60.5 to 62.9 years) and had longer diabetes duration (range of 19.4 to 20.7 years versus 10.5 to 15.1 years).

### Intervention

One parallel study utilized Ideg 200 units/mL (34) in the control arm and Igla-300 in the intervention arm, two parallel studies used Ideg 100 units/mL in the intervention arm and Igla-300 in the control arm (33, 39), and the concentration of Ideg was not defined in the cross-over study (40).

Both basal insulins were administered once daily in all trials. The timing of insulin administration varied across trials. Insulin was administered in the morning in one trial (39) (InRange trial); in the evening in one trial (33) (BRIGHT trial); at one of the following four time points (morning, at noon, in the evening, or at bedtime (40). The fourth trial (34) (CONCLUDE trial) randomized participants within each arm to administer basal insulin either in the morning (from waking to breakfast) or in the evening (from main evening meal to bedtime) at a 1:1 ratio and maintained the same dosing time throughout the trial.

Three trials included a titration phase (33, 34, 39) during which insulin doses were adjusted to reach target glycemic control. The titration phase ranged from approximately 8 weeks (39) (InRange trial) to 16 weeks (34) (CONCLUDE trial). The fourth trial evaluated glycemic targets during the last week of 4 weeks in a cross-over design (40).

Different insulin initiation regimens were applied as illustrated in the description of interventions (**Appendix 4**).

Background therapy consisted of oral hypoglycemic agents +/-GLP1RA in studies of type 2 diabetes and bolus insulin in studies of type 1 diabetes.

### Risk of bias in included studies

For the Cochrane RoB 2 assessment, we reviewed published reports (four trials), published protocols (four trials), data available in clinical trial registers (ClinicalTrials.gov for three studies and University Hospital Medical Information Network Clinical Trials Registry for one study), and statistical analysis plans (three trials, except Miura et al.). For each specific outcome, we established an overall ‘Risk of bias’ judgment, as well as judgments per ‘Risk of bias’ domain.

#### Change in HbA1c

**Figure 2:**
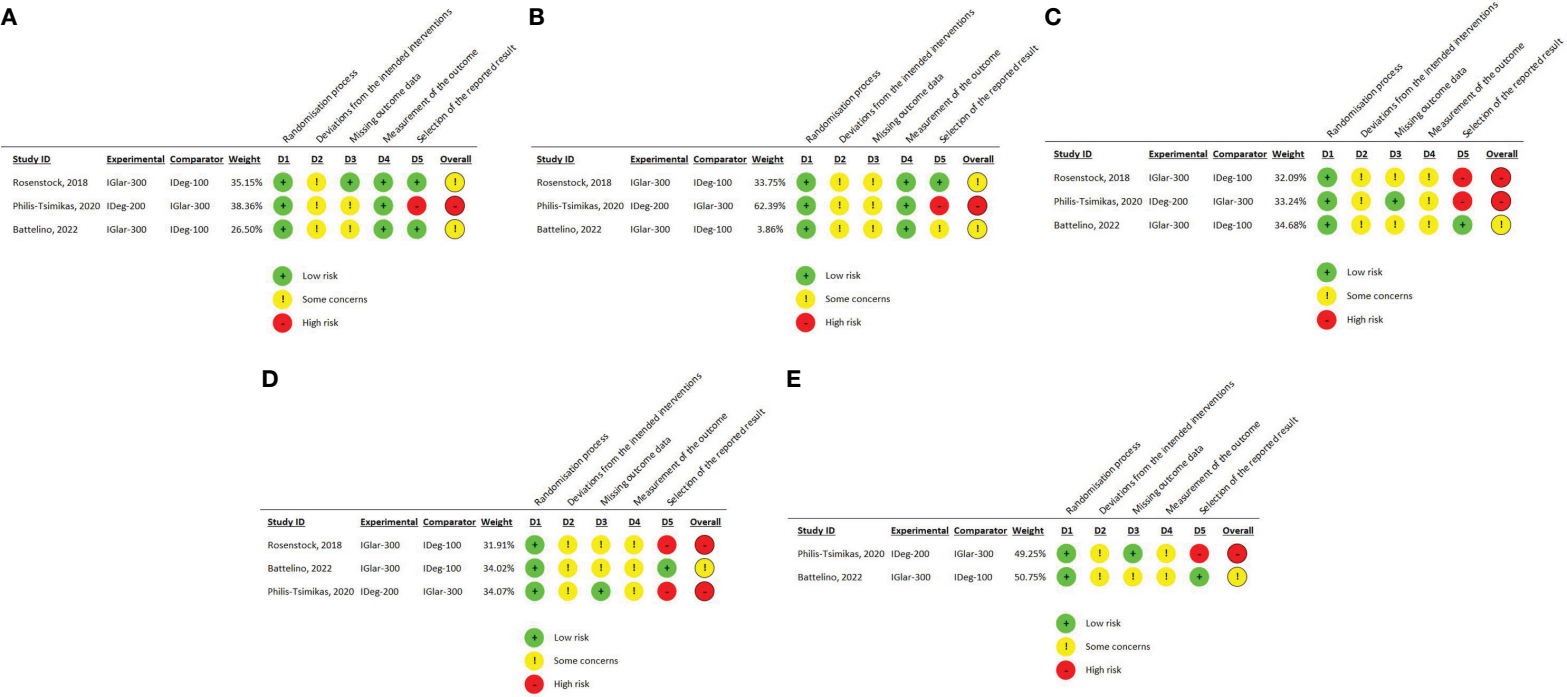
**(A)** RoB 2 traffic light plot for change in HbA1c **(B)** RoB 2 traffic light plot for change in FPG **(C)** RoB 2 traffic light plot for overall (anytime) hypoglycemia **(D)** RoB 2 traffic light plot for nocturnal hypoglycemia **(E)** RoB 2 traffic light plot for severe hypoglycemia.

One trial reporting change in HbA1c had a “high” overall risk of bias due to open-label design, protocol deviations (therapy discontinuation without provided justification), missing outcome data that was not accounted for by sensitivity analysis, and the *post hoc* analysis of the outcome (lack of pre-defined analysis plan for this outcome) (34). The remaining two trials had “some concerns” due to the open-label design, the exclusion of randomized patients who did not receive the allocated intervention from the ITT analysis (BRIGHT trial) (33), missing data, which was not accounted for by sensitivity analysis, and lack of justification for drug discontinuation (InRange) (39). RoB 2 traffic light plot for Change in HbA1c is summarized in **Figure 2A**.

#### Change in FPG

One trial reporting change in FPG had a high overall risk of bias due to the open-label design, protocol deviations due to therapy discontinuation without provided justification, missing outcome data that was not accounted for by sensitivity analysis, and the *post-hoc* analysis of the outcome (lack of pre-defined analysis plan for this outcome) (CONCLUDE trial) (34). Risk of bias in the remaining two trials was judged as “some concerns.” This was due to the open-label design, some randomized patients not receiving the allocated intervention, missing outcome data, and a lack of analysis methods to correct for bias of missing information (sensitivity analysis) (BRIGHT and InRange trials) (33, 39). In one trial, the outcome was not reported, as pre-planned in the study protocol, and was retrieved from ClinicalTrials.gov, which raised concerns about reporting risk of bias (39). RoB 2 traffic light plot for Change in FPG is summarized in **Figure 2B**.

#### Overall (anytime) hypoglycemia

We judged this outcome as “high risk of bias” in two studies (BRIGHT and CONCLUDE trials) (33, 34) and “some concerns” in the other two (39, 40).

All trials had open-label design and concerns about protocol deviations, analysis by “as-treated” instead of the ITT method, and concerns about missing outcome data that were not accounted for by sensitivity analysis.

The rating of this outcome as high risk was based mainly on the risk of reporting bias. In the BRIGHT trial (33), the protocol stated that hypoglycemia events will be categorized according to the American diabetes association (ADA) criteria (Severe, documented symptomatic, asymptomatic, probable symptomatic, pseudo hypoglycemia), whether confirmed by blood glucose or not, but reported only confirmed hypoglycemia. Additionally, in the CONCLUDE trial (34), this outcome was planned to be measured only during the maintenance phase (initial submission on ClinicalTrials.gov) but an amendment in 2020 included measurement during the whole treatment period. The statistical analysis plan and protocol were submitted after study completion. In the InRange trial (39), hypoglycemia encompassed both self-reported and SMBG-recorded events. Since this was an open-label study, the assessment of self-reported hypoglycemia could be potentially influenced by knowledge of the intervention received. The RoB 2 traffic light plot for overall (anytime) hypoglycemia is summarized in **Figure 2C**.

#### Nocturnal hypoglycemia

We judged this outcome as “high risk of bias” in two studies (BRIGHT and CONCLUDE trials) (34, 39) and “some concerns” in the third study (33). All trials had open-label design, two trials had concerns about protocol deviations (CONCLUDE and InRange trials) (34, 39), analysis by “as-treated” instead of ITT, and risk of bias arising from missing outcome data that were not accounted for by sensitivity analysis. In the InRange trial (39), hypoglycemia encompassed both self-reported and SMBG-recorded events. Since this was an open-label study, the assessment of self-reported hypoglycemia could be potentially influenced by knowledge of the intervention received.

The rating of this outcome as high risk was based on the risk of reporting bias. In the BRIGHT trial, the protocol stated that nocturnal hypoglycemia would be tested at the standard (00:00 to 05:59 AM) and extended (00:00 to 07:59 AM) periods. More hypoglycemic events were anticipated to occur during the extended period as both Igla-300 and Ideg reach maximal effect at this time. However, only confirmed hypoglycemia in the standard period was reported in the final study report. In the CONCLUDE trial (34), this outcome was planned to be measured only during the maintenance phase (initial submission on ClinicalTrials.gov), but an amendment in 2020 included measurement during the whole treatment period. The statistical analysis plan and protocol were submitted after study completion. The RoB 2 traffic light plot for nocturnal hypoglycemia is summarized in **Figure 2D**.

#### Severe hypoglycemia

We judged this outcome as “high risk of bias” in one trial (CONCLUDE trial) (34) and “some concerns” in one trial (InRange trial) (33). Both trials had open-label design and concerns about protocol deviations, analysis by “as-treated” instead of ITT, and concerns about missing outcome data that were not accounted for by sensitivity analysis. In the InRange trial (33), hypoglycemia encompassed both self-reported and SMBG-recorded events. Since this was an open-label study, the assessment of self-reported hypoglycemia could be potentially influenced by knowledge of the intervention received. In the CONCLUDE trial (34), this outcome was planned to be measured only during the maintenance phase (initial submission on ClinicalTrials.gov) but an amendment in 2020 included measurement during the whole treatment period. The statistical analysis plan and protocol were submitted after study completion. The RoB 2 traffic light plot for severe hypoglycemia is summarized in **Figure 2E**.

### Outcome measures

Three trials included a titration phase, during which insulin doses were adjusted to reach target glycemic control. The titration phase ranged from approximately 8 weeks (InRange trial) (39) to 16 weeks (CONCLUDE trial) (34). Two trials (BRIGHT and CONCLUDE) (33, 34) evaluated glycemic outcomes at the end of the titration and maintenance periods, and over the entire study duration. The third trial (InRange) (39) conducted end-of-study outcome assessments. The fourth trial (Miura et al.) (40), on the other hand, evaluated hypoglycemia over 1 week after 3 weeks of insulin titration and stabilization. Most insulin dose adjustments and reductions in glycemic measures occur during the titration period, a period that is followed by stabilization over the maintenance period. We evaluated primary and secondary outcomes at the end of the study period (overall duration), which is thought to be clinically relevant to routine care in which stable doses of basal insulin are administered for long-term diabetes treatment. A cutoff blood glucose level of <70 mg/dl (3.9 mmol/L) was chosen to define hypoglycemia. Guidelines of the ADA consider this cutoff to be clinically important regardless of the severity of acute hypoglycemic symptoms based on impaired counterregulatory responses to hypoglycemia and/or hypoglycemia unawareness experienced by many individuals with diabetes (23).

#### Change in HbA1c

**Figure 3:**
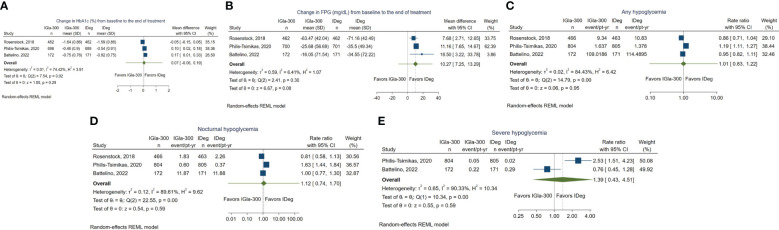
**(A)** Forest plot for change in HbA1c **(B)** Forest plot for change in FPG **(C)** Forest plot for anytime hypoglycemia. **(D)** Forest plot for nocturnal hypoglycemia. **(E)** Forest plot for severe hypoglycemia.

Three studies reported changes in HbA1c from baseline to end of treatment, as "least square mean difference of HbA1c" (33, 34, 39). Change in HbA1c (**Figure 3A**) was evaluated in 1442 patients in the Igla-300 group and 1439 patients in the IDeg group. HbA1c was analyzed in central certified laboratories in the three trials. There was no evidence of a difference in HbA1c (mean difference 0.07%, 95%CI -0.06-0.19; p=0.29; very low-certainty evidence [**Appendix 7**]). The 95% prediction interval was [-1.36 - 1.49], which is considered non-informative due to the small number of studies. We judged the overall risk of bias for this outcome as ‘high risk of bias’. The funnel plot and contour-enhanced funnel plot for HbA1c are reported in (**Appendix 5 – Figures 4A, B**). The Galbraith plot showed that none of the three trials is considered an outlier (**Appendix 6 –Figure 5A**).

#### Change in FPG

Three studies reported change in FPG from baseline, which was measured in central laboratories. One study reported the change in fasting glucose measured by SMBG. The changes in FPG were not reported in the published report of one trial (InRange) (39) and were retrieved from ClinicalTrials.gov.

Change in FPG (**Figure 3B**) was evaluated in 1442 participants in the Gla-300 group and 1439 participants in the IDeg group. There was a significant reduction in mean FPG from baseline in favor of IDeg (mean difference 10.27 mg/dL, 95% CI 7.25 to 13.29; P < 0.001; low-certainty evidence [**Appendix 7**]). The 95% prediction interval was [-11.59 - 32.12], which is considered non-informative due to the small number of studies. We judged the overall risk of bias for this outcome as ‘high risk of bias’. The funnel plot and contour-enhanced funnel plot for FPG are reported in (**Appendix 5 –Figures 4C, D**). The Galbraith plot showed that none of the three trials is considered an outlier (**Appendix 6 –Figure 5B**).

### Hypoglycemia

In three trials (33, 34, 39), hypoglycemia was estimated by incidence (percentage of participants with at least one hypoglycemic event) and event rate (events per patient time).

The estimation of hypoglycemia as event rate (events per person time) considers the risk of hypoglycemia recurrence during the on-treatment period and is comparable across variable study durations and person times. Thus, it was selected as a measure of hypoglycemia risk in preference over incidence (percentage).

One trial (Miura et al.) (40) reported the frequency of hypoglycemic events as a mean difference in the hypoglycemia rate between the two groups with 95% CI. However, the number of events was not reported. We contacted the primary author to retrieve the number of events in each group, but we did not receive a reply.

#### Anytime hypoglycemia

Hypoglycemia, occurring at anytime of the day (24 h), was reported in all studies. The BRIGHT trial (33) reported anytime confirmed hypoglycemia, defined as documented symptomatic or asymptomatic hypoglycemia (≤70 mg/dL or <54 mg/dL) and severe events, if any. The InRange trial (39), reported hypoglycemia of any category at anytime with plasma glucose levels <70 mg/dl, <70 and ≥54 mg/dl, and < 54 mg/dl. The CONCLUDE (34) defined overall symptomatic hypoglycemia as severe hypoglycemia (an event requiring third-party assistance) or confirmed (blood glucose <56 mg/dL (3.1 mmol/L) symptomatic hypoglycemic episodes. Miura et al. (40) reported a confirmed blood glucose level of <70 mg/dL [3.9 mmol/L] without a description of symptoms.

Among the three trials that reported the event rate of anytime hypoglycemia 1442 IGla-300 and 1493 IDeg users (**Figure 3C**), there was no evidence of a difference in the rate of hypoglycemia between the two groups (rate ratio 1.01, 95% CI 0.83 to 1.22, P = 0.95; very low-certainty evidence [Appendix 7]) (33, 34, 39). The 95% prediction interval was [0.1 - 10.63], which is considered non-informative due to the small number of studies. We judged the overall risk of bias for this outcome as ‘high risk of bias’.

The funnel plot and contour-enhanced funnel plot for anytime hypoglycemia are reported in (**Appendix 5 –Figures 4E, F**). The Galbraith plot showed that none of the three trials is considered an outlier (**Appendix 6 –Figure 5C**). Findings of the study by Miura et al. (40) demonstrated comparable frequency of hypoglycemic events (confirmed blood glucose of <70 mg/dL [3.9 mmol/L]) between the IGla-300 and IDeg groups (mean difference of 0.1 times per week, with a 95% CI of −0.2 to 0.3 times per week, P = 0.54).

#### Nocturnal hypoglycemia

Three trials reported nocturnal hypoglycemia (33, 34, 39), which was defined as hypoglycemia that occurred during the standardized period (00:00 and 05:59 AM). The glucose cutoffs used to define nocturnal hypoglycemia varied between trials.

The BRIGHT trial (33) reported plasma glucose ≤70 mg/dL (≤3.9 mmol/L), or < 54 mg/dL (<3.0 mmol/L)); the InRange trial (39) reported blood glucose < 70, < 70, and ≥ 54 mg/dl, or < 54 mg/dl; and the CONCLUDE reported blood glucose <56 mg/dL (<3.1 mmol/l). One study reported confirmed hypoglycemia (33), another reported any, severe, and/or confirmed hypoglycemia (39), and the third study reported only severe or symptomatic nocturnal hypoglycemia (34).

Among the 1442 IGla300 and 1493 IDeg treated participants (**Figure 3D**), there was no evidence of statistically significant difference in the event rate of nocturnal hypoglycemia (Rate Ratio 1.12; 95% CI 0.74 to 1.70); P = 0.59; very low-certainty evidence [**Appendix 7**]). The 95% prediction interval was [0.01 - 191.18], which is considered non-informative due to the small number of studies contributing to this outcome. We judged the overall risk of bias for this outcome as ‘high risk of bias’. The funnel plot and contour-enhanced funnel plot for nocturnal hypoglycemia are reported in (**Appendix 5 –Figures 4G, H**). The Galbraith plot showed that none of the three trials is considered an outlier (**Appendix 6 –Figure 5D**).

#### Severe hypoglycemia

Severe hypoglycemia was defined in accordance with ADA guidelines (23) as an event in which the participant required the assistance of another person to actively administer carbohydrate, glucagon, or other resuscitative actions. The definition was consistent across all trials.

The effect estimate of severe hypoglycemia was pooled for two trials only (34, 39) (**Figure 3E**). In one trial, only one episode of hypoglycemia was reported in the IGla-300 group (34).

As person time in the trial was not reported, calculation of the rate ratio was unfeasible. In the fourth trial, no severe hypoglycemia events were reported in either group.

Among the 976 IGla300 and 976 IDeg treated patients, there was no evidence of a statistically significant difference in the event rate of severe hypoglycemia (Rate Ratio 1.39; 95% CI 0.43 to 4.51; P = 0.59; very low-certainty evidence [**Appendix 7**]). The 95% prediction interval could not be generated as only two studies contributed to this outcome. We judged the overall risk of bias for this outcome as ‘high risk of bias’. The funnel plot and contour-enhanced funnel plot for severe hypoglycemia are reported in (**Appendix 5 –Figures 4I, J**). The Galbraith plot showed that none of the three trials is considered an outlier (**Appendix 6 –Figure 5E**).

## Discussion

The current review is the first to compare IGla-300 exclusively to IDeg. We reviewed four head-to-head randomized controlled trials (33, 34, 39, 40) including a total of 2927 patients; 1442 participants were randomized to IGla-300, 1439 participants were randomized to IDeg, and 46 were enrolled in a cross-over trial.

Overall, there was no evidence of difference between the two insulins in the mean change in HbA1c and the risk of anytime, nocturnal, and severe hypoglycemia. IDeg appeared to cause a higher reduction in FPG compared to IGla-300. However, considering the heterogeneity and high risk of bias, these findings should be interpreted with caution.

Our findings of comparable reduction in HbA1c and severe hypoglycemia and significantly lower FPG with IDeg compared to IGla-300 are in line with those previously reported in the review by Dong et al., which compared IDeg to all formulations of insulin glargine and insulin detemir collectively. Yang et al. (21) also reported significantly higher reductions in FPG with IDeg compared to combined IGla-100 and IGla-300.

It is worth mentioning that trials comparing IGla-300 and IDeg in individuals with type 2 diabetes excluded patients receiving bolus insulin; thus, the extent to which their findings could be generalized to those groups of patients is questionable. Moreover, these findings may not be generalizable to patients with advanced kidney disease as they were excluded from the trials. The currently ongoing TRENT trial (41) (Gla-300 and IDeg-100 in insulin-naïve people with type 2 diabetes mellitus and renal impairment, NCT05552859) is expected to provide valuable insights into the use of these agents in patients with diabetes and chronic kidney disease. Hospitalized patients were also excluded from our review. Further understanding of the efficacy and safety of those agents in the hospital setting is warranted.

The main strength of our review is being the first to provide a direct head-to-head comparison between second-generation ultra-long-acting basal insulin analogs. Previously published systematic reviews (20–22) presented pooled estimates of comparisons between IDeg and insulin glargine alone (regardless of concentration) or along with other basal insulins. In view of the evidence of variations in PK/PD and clinical outcomes between IGla-300 and IGla-100, a direct head-to-head comparison is important to inform decision-making. Other strengths include the comprehensive systematic review of literature without language restriction and the inclusion of the latest type 1 and type 2 diabetes trials.

### Limitations

The current review has some limitations. First, all trials had open-label design, which could have affected the titration of insulin doses and self-reported hypoglycemia episodes. Nonetheless, this was partly accounted for by reporting blood glucose-confirmed hypoglycemia in all trials but one. Second, our findings demonstrated significantly high heterogeneity across all outcomes, except mean change in FPG. The low heterogeneity detected with FPG should be interpreted with caution as only three studies contributed to this outcome.

The high heterogeneity reported in this review could be explained by the clinical and methodological heterogeneity of the trials. Enrolled patients had variable type and duration of diabetes, baseline HbA1c, age, background therapies, and risk of hypoglycemia. Moreover, hypoglycemia definitions, insulin titration protocols, and timings of outcome assessments were inconsistent across trials. Additionally, all outcomes evaluated had “high” or “some concerns” risk of bias, and all included trials were funded by manufacturers of IDeg and Gla-300.

A previous Cochrane review raised concerns about bias introduced by the broad range of interventions encompassed within the definition of third-party assistance in severe hypoglycemia (e.g., administration of food, drink, parenteral subcutaneous glucagon injection, or intravenous glucose), which is a limitation to be addressed by future research (20).

The small number of studies evaluated in the current review limited our ability to perform many planned tests. These include the quantification of heterogeneity by Egger’s test, subgroup analysis to investigate the effect of the type of diabetes on outcomes, and the assessment of publication bias and small study effects by funnel plot and contour-enhanced funnel plots. The decreased precision of the summary effects is another limitation that is attributed to the number of studies.

## Conclusion

The current review demonstrated a lack of evidence of a difference between the IGla-300 and IDeg in the mean change in HbA1c and risk of anytime, nocturnal, and severe hypoglycemia. IDeg appeared to cause a higher reduction in FPG compared to IGla-300. These findings should be interpreted with caution considering the imprecision, high heterogeneity, and risk of bias. The selection between the two agents should consider other factors such as cost and patient preference. If these factors are deemed similar in a particular setting, clinicians may consider choosing IDeg in a subset of patients with uncontrolled FPG. Further studies with robust methodologies and standardized outcome definitions are needed to further ascertain the differences between these two agents.

## Data availability statement

The original contributions presented in the study are included in the article/**Supplementary Material**. Further inquiries can be directed to the corresponding author.

## Author contributions

EA: Conceptualization, Data curation, Formal Analysis, Investigation, Methodology, Project administration, Resources, Software, Validation, Visualization, Writing – original draft, Writing – review & editing. MS: Conceptualization, Data curation, Formal Analysis, Investigation, Methodology, Project administration, Resources, Software, Validation, Visualization, Writing – original draft, Writing – review & editing. NO: Conceptualization, Data curation, Formal Analysis, Funding acquisition, Investigation, Methodology, Project administration, Resources, Software, Validation, Visualization, Writing – original draft, Writing – review & editing.
