# Supplementary material for: Efficacy and safety of insulin glargine 300 units/mL vs insulin degludec in patients with type 1 and type 2 diabetes: a systematic review and meta-analysis
Source: Front Endocrinol (Lausanne). 2024 Jan 19;14:1285147. doi: 10.3389/fendo.2023.1285147 (PMC10836592; doi:10.3389/fendo.2023.1285147)
Supplement: Supplementary file 1 [file DataSheet_1.docx]

# Appendix 1: Details of search strategy

## PubMed

| Search number | Query | Results |
| --- | --- | --- |
| 1 | glargin*[Title/Abstract] | 3,034 |
| 2 | Toujeo[Title/Abstract] | 21 |
| 3 | Gla-300[Title/Abstract] | 168 |
| 4 | IGlar-300[Title/Abstract] | 5 |
| 5 | "Insulin Glargine"[MeSH Terms] | 2,157 |
| 6 | #1 OR #2 OR #3 OR #4 OR #5 | 3,386 |
| 7 | degludec[Title/Abstract] | 766 |
| 8 | Tresiba[Title/Abstract] | 27 |
| 9 | IDeg[Title/Abstract] | 178 |
| 10 | "insulin degludec" [Supplementary Concept] | 411 |
| 11 | #7 OR #8 OR #9 OR #10 | 816 |
| 12 | diabet*[Title/Abstract] | 742,110 |
| 13 | "Diabetes Mellitus"[Mesh] | 488,555 |
| 14 | T1D[Title/Abstract] | 9,178 |
| 15 | T2D[Title/Abstract] | 14,764 |
| 16 | IDDM[Title/Abstract] | 6,902 |
| 17 | NIDDM[Title/Abstract] | 6,974 |
| 18 | #12 OR #13 OR #14 OR #15 OR #16 OR #17 | 803,230 |
| 19 | randomized controlled trial [Publication Type] | 579,215 |
| 20 | controlled clinical trial[Publication Type] | 669,368 |
| 21 | Randomiz*[Title/Abstract] | 629,447 |
| 22 | randomis*[Title/Abstract] | 124,827 |
| 23 | randomly[Title/Abstract] | 393,443 |
| 24 | trial[Title/Abstract] | 728,342 |
| 25 | #19 OR #20 OR #21 OR #22 OR #23 OR #24 | 1,585,620 |
| 26 | #6 AND #11 AND #18 AND #25 | 215 |

## Embase

| No. | Query Results | Results |
| --- | --- | --- |
| #1. | glargin*:ti,ab,kw | 6,329 |
| #2. | toujeo:ti,ab,kw | 54 |
| #3. | 'gla-300':ti,ab,kw | 439 |
| #4. | 'iglar-300':ti,ab,kw | 3 |
| #5. | 'insulin glargine'/exp | 11,896 |
| #6. | #1 OR #2 OR #3 OR #4 OR #5 | 12,746 |
| #7. | degludec:ti,ab,kw | 1,722 |
| #8. | tresiba:ti,ab,kw | 62 |
| #9. | ideg:ti,ab,kw | 618 |
| #10. | 'insulin degludec'/exp | 2,003 |
| #11. | #7 OR #8 OR #9 OR #10 | 2,474 |
| #12. | diabet*:ti,ab,kw | 1,137,334 |
| #13. | 'diabetes mellitus'/exp | 1,181,794 |
| #14. | t1d:ti,ab,kw | 18,749 |
| #15. | t2d:ti,ab,kw | 26,942 |
| #16. | iddm:ti,ab,kw | 8,156 |
| #17. | niddm:ti,ab,kw | 8,285 |
| #18. | #12 OR #13 OR #14 OR #15 OR #16 OR #17 | 1,385,231 |
| #19. | [randomized controlled trial]/lim | 733,373 |
| #20. | 'controlled clinical trial'/de | 438,212 |
| #21. | randomiz*:ti,ab,kw | 929,230 |
| #22. | randomis*:ti,ab,kw | 186,188 |
| #23. | randomly:ti,ab,kw | 520,863 |
| #24. | trial:ti,ab,kw | 1,067,183 |
| #25. | #19 OR #20 OR #21 OR #22 OR #23 OR #24 | 2,254,088 |
| #26. | #6 AND #11 AND #18 AND #25 | 684 |

## Cochrane Central Register of Controlled Trials (CENTRAL)

ID Search

#1 (glargin*):ti,ab,kw (Word variations have been searched)

#2 (toujeo):ti,ab,kw (Word variations have been searched)

#3 MeSH descriptor: [Insulin Glargine] this term only

#4 (Gla-300):ti,ab,kw (Word variations have been searched)

#5 (IGlar-300):ti,ab,kw (Word variations have been searched)

#6 #1 OR #2 OR #3 #4 OR #5

#7 (DEGLUDEC):ti,ab,kw (Word variations have been searched)

#8 (IDeg):ti,ab,kw (Word variations have been searched)

#9 (tresiba):ti,ab,kw (Word variations have been searched)

#10 #7 OR #8 OR #9

#11 (diabet*):ti,ab,kw (Word variations have been searched)

#12 MeSH descriptor: [Diabetes Mellitus] explode all trees

#13 (T1D):ti,ab,kw (Word variations have been searched)

#14 (T2D):ti,ab,kw

#15 (IDDM):ti,ab,kw

#16 (NIDDM):ti,ab,kw

#17 #11 OR #12 OR #13 OR #14 OR #15 OR #16

#18 #6 AND #10 AND #17

# Appendix 2: The process of data collection and analysis

1. Data extraction
2. Assessment of risk of bias in included studies
3. Measurement of treatment effects
4. Dealing with missing data
5. Data synthesis
6. Assessment of heterogeneity
7. Subgroup analysis and investigation of heterogeneity
8. Assessment of reporting bias
9. Assessment of certainty of evidence

# Appendix 3: Characteristics of included studies

Studies ordered according to year of publication

**Rosenstock J (BRIGHT trial), 2018**

| Study characteristics | |
| --- | --- |
| Methods | Design: a multicenter, open-label, 1:1 randomized, active-controlled, 2-arm, parallel-group, 24-week treatment duration Phase 4 study |
| Participants | Inclusion criteria:   - Adult participants (18 Years and older) with type 2 diabetes mellitus inadequately controlled with OADs therapy with/without GLP-1 receptor agonist at stable dose for at least 3 months. - Signed written informed consent.   Exclusion criteria:   - Age <18 years. - HbA1c <7.5% or >10.5% (at screening visit). Body mass index (BMI) <25 kg/m^2 or >40 kg/m^2. - History of type 2 diabetes for less than 1 year before screening. - Less than 6 months before screening on OADs treatment and GLP-1 receptor agonist (if taken). - Current or previous insulin use except for a maximum of 8 consecutive days or totally 15 days (e.g., acute illness, surgery) during the last year prior to screening. - Initiation of new glucose-lowering medications and/or weight loss drug in the last 3 months before screening visit. - Participant receiving only noninsulin antihyperglycemic drugs not approved for combination with insulin according to local labelling/local treatment guideline. - History of hypoglycemia unawareness or repeated episodes of severe hypoglycemia or metabolic acidosis, including hospitalization for diabetic ketoacidosis during the last 12 months prior to screening. - Unstable proliferative diabetic retinopathy or any other rapidly progressive diabetic retinopathy or macular edema likely to require treatment (e.g., laser, surgical treatment, or injectable drugs) during the study period. - End stage renal disease. - Any acute or chronic condition that in the opinion of Investigator would affect the safety of participant, compliance, or study results. - Any contraindication to use of Toujeo® or Tresiba® as defined in the national product label, hypersensitivity to Toujeo® or Tresiba® active ingredients or one of the excipients. - Pregnant or breast-feeding women.   Diagnostic criteria: Quote “It will be the investigator’s responsibility to confirm the diagnosis of type 2 diabetes”  Number of study centers: (158 sites, 16 countries: US, Europe and Israel) |
| Interventions | Intervention(s): Insulin glargine, 300 units/mL  Comparator(s): Insulin degludec, 100 units/mL  Duration of intervention: 24 weeks  Duration of follow-up: 24 weeks  Run-in period: — |
| Outcomes | Primary Outcome:   - Change from baseline in HbA1c to Week 24   Secondary outcomes:   - Change from baseline in HbA1c to week 12. - Change From baseline in Fasting Plasma Glucose (FPG) and Fasting Self-Monitoring Plasma Glucose (SMPG) to Week 12 and Week 24  Change from baseline in 8 point and 4-point SMPG profile to week 12 and Week 24 per time point - Change from baseline in 24-hour average 8-point SMPG profile to week 12 and week 24 - Change from baseline in variability of fasting SMPG and 24-Hour 8-point SMPG profiles to week 12 and week 24 - Percentage of participants reaching target HbA1c of < 7% and =<6.5% at week 12 and week 24 (with and without severe and/or confirmed hypoglycemia (70 mg/dL) Event) - Percentage of Participants with sulphonyl urea or meglitinide dose reduction/ discontinuation due to hypoglycemia - Percentage of participants requiring a rescue therapy during 24 weeks treatment period - Change from baseline in basal insulin dose (U/kg Body Weight) to week 12 and week 24   -Hypoglycemia (any, severe and/or confirmed hypoglycemia: any time of the day and nocturnal):  event rate per participant year during and percentage of participants with at least one hypoglycemic even [Time Frame: day 1-week 12, week 13-week 24, and 24week period] |
| Study registration | Trial identifier: NCT02738151; 2015-005101-36 (EudraCT Number),  U1111-1177-6327 (another identifier: UTN)  Study terminated early: no |
| Publication details | Language of publication: English  Funding: commercial funding (Sanofi)  Publication status: peer-reviewed journal |
| Stated aim of study | Quote: " To compare insulin glargine 300 units/mL (Gla-300) versus insulin degludec 100 units/mL (IDeg-100) in this first head-to-head randomized controlled trial. |
| Notes | Number of events of hypoglycemia was not reported, only event rate was reported  Does not  The study objective stated “To assess the frequency of occurrence and diurnal distribution of hypoglycemia by category of hypoglycemia (symptomatic, asymptomatic, nocturnal, severe, probable and pseudo)” but only confirmed hypoglycemia was reported  Four authors are employees/shareholders of Sanofi (funder)  Change in basal insulin dose was also assessed, although this was not a prespecified end point. At week 24 the Gla-300 insulin dose was higher by 0.11 units/kg than the IDeg-100 dose, an increase in the mean dose difference by 0.04 units/ kg compared with baseline.  Editorial and writing assistance was funded by Sanofi.  Sanofi sponsored, designed, and coordinated the clinical trial  Study authors declared receiving honoraria or consulting fees from Sanofi (the funder) |

**Philis-Tsimikas A (CONCLUDE trial), 2020**

| Study characteristics | |
| --- | --- |
| Methods | Design: a multicenter, open-label, 1:1 randomized, active-controlled, 2-arm, parallel-group, 52-week planned treatment duration (extended to a maximum of 88 weeks by a protocol amendment) phase 4 study. |
| Participants (post-amendment) | Inclusion criteria:   - Male or female, age above or equal to 18 years at the time of signing informed consent. - HbA1c ≤9.5% - Body mass index ≤45 kg/m2 - Subjects fulfilling at least one of the below criteria: - Experienced at least one severe hypoglycemic episode within the last year (according to the ADA definition, April 2013b). - Moderate chronic renal failure, defined as glomerular filtration rate 30 - 59 mL/min/1.73 m2 per CKD-EPI by central laboratory analysis. - Hypoglycemic symptom unawareness. - Treated with insulin for more than 5 years. - Episode of hypoglycemia (defined by symptoms of hypoglycemia and/or episode with low glucose measurement (≤ 70 mg/dL [≤ 3.9 mmol/L])) within the last 12 weeks prior to Visit 1(screening). - Subjects diagnosed (clinically) with type 2 diabetes mellitus. - Treated with basal only insulin (once daily or twice-daily insulin (insulin detemir; insulin glargine 100 units/mL, biosimilar of insulin glargine 100 units/mL or insulin Neutral Protamine Hagedorn)) ≥ 90 days prior to the day of screening with or without any of the following anti-diabetic drugs with stable doses for ≥ 90 days prior to screening:   a Metformin b Dipeptidyl peptidase -4 inhibitor c Sodium-glucose co-transporter 2 inhibitor d Alpha-glucosidase-inhibitors (acarbose) e Thiazolidinediones  Key exclusion criteria   - Treatment with bolus or premixed insulin, continuous subcutaneous insulin infusion, glucagon-like peptide 1 receptor agonists, or sulphonylureas/glinides within 90 days before the screening visit - Severe renal impairment (estimated glomerular filtration rate <30 mL/min/1.73m2) - Impaired liver function (alanine aminotransferase or aspartate transaminase ≥2.5 times upper limit of normal at screening)   Number of study centers: (229 sites in 11 countries in US and Europe) |
| Interventions | Intervention(s): Insulin glargine, 300 units/mL  Comparator(s): Insulin degludec, 200 units/mL  Duration of intervention: Planned: 52 weeks (the first 16 weeks for titration), After amendment: up to 88 weeks (the first 16 weeks for titration)  Duration of follow-up: 4 weeks after the end of intervention  Screening/randomization period: 2 weeks before the intervention |
| Outcomes (post-amendment) | Primary Outcome:   - Number of severe or BG-confirmed symptomatic hypoglycemic episodes during maintenance period 2   Secondary outcomes:   - Basal insulin dose (U) at end of maintenance period 2 - Number of nocturnal, severe or BG-confirmed symptomatic hypoglycemic episodes during maintenance period 2 - Number of severe hypoglycemic episodes during maintenance period 2 - Change from baseline to end of maintenance period 2 in HbA1c, FPG, SMBG, and body weigh - Number of severe or BG-confirmed symptomatic hypoglycemic episodes from randomization to end of maintenance period 2 - Number of nocturnal, severe or BG-confirmed symptomatic hypoglycemic episodes from randomization until end of maintenance period 2 - Number of severe hypoglycemic episodes from randomization until end of maintenance period 2 - Number of adverse events from randomization to end of maintenance period 2 |
| Study registration | Trial identifier: NCT03078478  Other Study ID Numbers:  NN1250-4252  U1111-1184-8175 (Other Identifier: WHO)  2016-002801-20 (EudraCT Number)  Study terminated early: no |
| Publication details | Language of publication: English  Funding: commercial funding (Novo Nordisk)  Publication status: peer-reviewed journal |
| Stated aim of study | to investigate the effect of insulin degludec 200 U/ml (degludec U200) and glargine U300 on hypoglycemia in insulin-treated individuals with type 2diabetes. |
| Notes | Number of events of hypoglycemia and event rate were reported.  The trial was amended as the glucometers routine monitoring of blinded data showed an unusual pattern in the reporting of glycemic variables and hypoglycemic events. Glycemic data were inconsistent between central-laboratory-measured variables (HbA1cand fasting plasma glucose [FPG]) and patient-reported fasting self-measured blood glucose (SMBG) values. Data available from SMBG monitoring indicated to the patient that the blood glucose levels were higher than they were, potentially increasing the risk of hypoglycemia because of unnecessary insulin up-titration. At the time of the amendment, the number of patient-reported hypoglycemic events confirmed by blood glucose was low while the number of pseudo-hypoglycemic events (blood glucose >3.9 mmol/l with symptoms) was high.  Novo Nordisk was involved in the design of the trial, the collection and analysis of data, and writing the clinical report. |

**Miura H, 2020:**

| Study characteristics | |
| --- | --- |
| Methods | Design: randomized, crossover, open-label, parallel-group, multicenter study |
| Participants | Inclusion criteria:   - Individuals with type 1 diabetes, aged ≥20 years, serum C-peptide immunoreactivity is < 0.2 ng/mL (as confirmed at least twice) - Treatment for at least 1 year with basal-bolus insulin injections, with IGla-100, IGla-300, or IDeg as the basal insulin and a rapid-acting insulin analogue or regular insulin as the bolus insulin. - Ability to perform SMBG and FGM   Exclusion criteria:   - HbA1c level of 9.0% or higher - Use of medications that affect glucose metabolism (such as betablockers, corticosteroids, and monoamine oxidase inhibitors) - History of myocardial infarction, angina, coronary bypass surgery, or Heart failure within the previous 6 months - severe hypertension (systolic blood pressure of at least 180 mmHg or diastolic blood pressure of at least 100 mmHg) - Severe liver dysfunction (serum aspartate aminotransferase or alanine - Aminotransferase levels of at least 2.5 times the upper limit of normal) - Severe renal impairment (serum creatinine concentration of at least 2.0 mg/dL) - Frequently recurring severe hypoglycemia or hospitalization because of - Serious hypoglycemia or diabetic ketoacidosis within the previous year - Proliferative diabetic retinopathy with a high risk of hemorrhage - Existing or possible pregnancy or breastfeeding or no use of an adequate contraceptive method (adequate contraceptive measures as recommended by local regulation or practice) - A diagnosis of cancer - A complicating psychiatric disorder - Alcoholism or another drug addiction - Diabetes other than type 1, or type 1 diabetes with - Remaining insulin secretory capacity - Rejection of SMBG or FGM - Declaration by an investigator that the individual is otherwise inappropriate for the study.   Diagnostic criteria: Quote “type 1 diabetes whose fasting C-peptide levels were essentially zero (confirmed to be <0.2 ng/mL at least twice)”  Number of study centers: (13 reported in protocol, 14 in final report, in Japan) |
| Interventions | Intervention(s): Insulin glargine, 300 units/mL  Comparator(s): Insulin degludec (concentration not defined)  Duration of intervention: 4 weeks-switch-4 weeks (total 8 weeks)  Duration of follow-up: 12 weeks  Run-in period: — |
| Outcomes | Primary Outcome   - Day to-day variability of FPG level as evaluated by the SD of the plasma glucose concentration determined by SMBG before breakfast during the last week of each 4-week treatment period   Secondary outcomes:   - The coefficient of variance for the plasma glucose level determined by SMBG before breakfast during the last week of each 4-week treatment   period as an indicator of inter-day glycemic variability independent of FPG level itself   - The intraday glycemic variability calculated from the seven daily measurements of plasma glucose by SMBG or from FGM data during the last week of each treatment period - The frequency of hypoglycemic events - The duration of hypoglycemia determined by FGM - The administered basal and bolus insulin dose (the mean for the last week of each treatment period). |
| Study registration | Trial identifier:  Japan Registry of Clinical Trials (jRCTs051180138), University Hospital Medical Information Network Clinical Trials Registry (UMIN000029630).  Study terminated early: no |
| Publication details | Language of publication: English  Funding: commercial funding (Novo Nordisk Pharma Ltd.)  Publication status: peer-reviewed journal |
| Stated aim of study | Quote: " The primary aim of the study was evaluation of the non-inferiority of  IDeg relative to IGla-300 in terms of day-to-day variability of FBG  levels as evaluated by the standard deviation (SD) determined from  SMBG data”. |
| Notes | Baseline characteristics is presented as cumulative for all patients and not listed for each treatment group.  The frequency of hypoglycemic events was reported ad mean difference of episodes per week, with a 95% CI of −0.2 to 0.3 times per week, no of events and rate per group was not reported  Authors received research support and lecture fees from Novo Nordisk (the funder and manufacturer of IDeg)  The company had no role in the design and conduct of the study; in the  collection, management, analysis and interpretation of the data; or in  the preparation, review or approval of the manuscript. |

**Battelino T (InRange trial), 2022**

| Study characteristics | |
| --- | --- |
| Methods | Design: A multicenter, randomized, active-controlled, parallel-group, 12-week, open-label, Phase IV study collected blinded CGM data over 20 consecutive days from adults with type 1 diabetes.  Participants were randomized to receive Insulin glargine 300 or insulin degludec 100 units/mL once per day. |
| Participants | Inclusion criteria:   - Participants with Type 1 Diabetes mellitus. - Participants treated with multiple daily injections using basal insulin analog once daily and rapid acting insulin analogs for at least one year. - HbA1c greater than or equal to (>=) 7 percent (%) (53 millimoles per mole [mmol/mol]) and less than or equal to (<=) 10% (86 mmol/mol) at screening.   Exclusion criteria:   - Participants not on stable dose of basal insulin analog. - Participants having received Toujeo or Tresiba as basal insulin within 30 days prior to screening. - Participants not having used the same insulins (both basal and rapid) within 30 days prior to screening. - Participants having received basal insulin dose >= 0.6 units per kilogram body weight within 30 days prior to screening. - Participants having received any glucose lowering drugs (including any premixed insulins, human regular insulin as mealtime insulins, any others injectable or oral), other than basal and rapid insulin analogs, within 3 months prior to screening. - End stage renal disease or on renal replacement treatment. - Retinopathy or maculopathy with one of the following treatments, either recent (within 3 months prior to screening) or planned: intravitreal injections or laser or vitrectomy surgery. - Body weight change >=5 kilogram within 3 months prior to screening. |
| Interventions | Intervention(s): Drug: Insulin glargine, 300 U/mL  Comparator(s): Insulin degludec, 100 units/mL  Background therapy in both arms: Rapid acting insulin analogs  The duration of the study per participant was around 18 weeks:  1 or 2 weeks of screening  Followed by a 4-week run-in period,  Followed by a 12-week treatment period and a 2 to 4 days follow-up period.  Following an 8-week titration period with Gla-300 or IDeg-100, continuous glucose monitoring (CGM) device was given to participants in week 9 and endpoint CGM data was collected over 20 consecutive days during weeks 10–12 |
| Outcomes | Primary outcome:   - Percentage of time of glucose concentration within the target range of greater than or equal to (>=) 70 to less than or equal to (<=) 180 milligrams per deciliter: non-inferiority analysis [ time frame: during week 10 up to week 12]   Secondary outcomes:   - Glucose total coefficient of variation (cv%) [ time frame: during week 10 up to week 12] - Percentage of time of glucose concentration within the target range of >=70 to <=180 milligrams per deciliter: superiority analysis [ time frame: during week 10 up to week 12] - Glucose within-day cv% and between-day cv% [ time frame: during week 10 up to week 12] - Change from baseline in glycated hemoglobin a1c (hba1c) at week 12 [ time frame: baseline, week 12] - Change from baseline in fasting plasma glucose (FPG at week 12 [ time frame: baseline, week 12] - Percentage of time with glucose level <70 milligrams per deciliter (all time and during the night) [ time frame: during week 10 up to week 12] - Mean hours per day with glucose level <70 milligrams per deciliter (all time and during the night) [ time frame: during week 10 up to week 12] - Percentage of time with glucose level >180 milligrams per deciliter [ time frame: during week 10 up to week 12] - Mean hours per day with glucose level >180 milligrams per deciliter [ time frame: during week 10 up to week 12] - Number of participants with at least one hypoglycemic event during the on-treatment period [ time frame: from the first injection of imp up to 2 days after the last injection of imp (i.e., up to 86 days)] - Number of hypoglycemic events per participant year during the on-treatment period [ time frame: from the first injection of imp up to 2 days after the last injection of imp (i.e., up to 86 days)] |
| Study registration | Trial identifier: [NCT04075513](https://clinicaltrials.gov/show/NCT04075513) ; Other Study ID Numbers: LPS14947 2017-002756-91 (EudraCT Number) U1111-1197-8171 (Other Identifier: UTN)  Study terminated early: no |
| Publication details | Language of publication: English  Funding: commercial funding (Sanofi)  Publication status: peer-reviewed journal |
| Stated aim of study | To demonstrate that Glargine 300 is noninferior to insulin degludec 100 units/mL in terms of glycemic control, measured by time-in-range (TIR) and variability, as assessed using CGM, in adults with type 1 diabetes |
| Notes | It is important to note that blinded CGM allows the capture of CGM parameters without the participant’s behavior being influenced by their own CGM-derived glucose readings.  This is particularly relevant in open-label treatment trials where knowledge of both the treatment and the measured outcome could lead to bias in the results.4 CGM data did not guide insulin titration. Instead, basal insulin was titrated to achieve the target fasting SMPG of ≥3.9 to <5.6 mmol/L while avoiding hypoglycemic episodes.  Funded by intervention arm manufacturer (Sanofi).  The role of the sponsor was not mentioned.  Editorial Assistance was used and was funded by Sanofi.  Conflict of interests was declared.  Most of authors either service as advisory board member for samosa, received honorarium or research grant from Sanofi.  Three authors are employees and shareholders of Sanofi S.A. |

FGM: Flash glucose monitoring; SMBG : Self-monitoring of blood glucose

# Appendix 4: Description of interventions (studies ordered according to year of publication)

| Rosenstock J (BRIGHT trial), 2018 | |
| --- | --- |
| Intervention | Experimental:  Toujeo® (Insulin glargine, 300 units/mL) subcutaneous (SC) injection once daily in the evening using a pre-filled pen, up to Week 24 on top of non-insulin antidiabetic treatment.  Dose titration to achieve fasting self-monitored plasma glucose (SMPG) from 80 to 100 mg/dL (4.4 to 5.6 mmol/L).  Other Names: HOE901-U300  Background therapy: Oral Anti diabetics Drugs (OADs), Glucagon-like peptide-1 (GLP-1) receptor agonist.  Active Comparator:  Tresiba® (Insulin Degludec, 100 units/mL) subcutaneous (SC) injection once daily in the evening using a pre-filled pen, up to Week 24 on top of non-insulin antidiabetic treatment  Dose titration to achieve fasting self-monitored plasma glucose (SMPG) from 80 to 100 mg/dL (4.4 to 5.6 mmol/L).  Other Name: Tresiba  Background therapy: Oral Anti diabetics Drugs (OADs), Glucagon-like peptide-1 (GLP-1) receptor agonist. |
| Titration period | 12 weeks |
| Strength of insulin | 100 units/mL degludec, 300 units/mL glargine |
| Concomitant medications | Oral Anti diabetics Drugs (OADs), Glucagon-like peptide-1 (GLP-1) receptor agonist. |
| Glycemic targets | Quote” a target fasting SMPG of 80 to 100 mg/dL (4.4 to 5.6 mmol/L) while avoiding hypoglycemia episodes.” |
| Interval of blood glucose  measurement |  |
| Calibration of blood  glucose measurement  device | Quote “The glucometers should be calibrated according to instructions given in the package leaflet and the study site should also check the glucometers regularly using the provided control solutions for data validity.” |
| Adjusting insulin doses | Dose adjustment algorithm   \| Median of fasting SMBG (mg/dL) from the last 3 measurements \| Dose change \| \| --- \| --- \| \| >140 mg/dl (7.8 mmol/L) \| + 6 U \| \| >120 and ≤ 140 (>6.7 and ≤7.8 mmol/L) \| + 4 U \| \| >100 and ≤120 (>5.6 and ≤ 6.7 mmol/L) \| + 2 U \| \| ≥80 and ≤100 (≥4.4 and ≤ 5.6 mmol/L) \| No change \| \| <80(4.4 mml/L) or occurrence of 1 symptomatic confirmed hypoglycemia episode documented in the preceding week \| -2U or at the discretion of the investigator \| |
| Interval for insulin adjustments | Quote” After randomization, the dose will be titrated at least weekly (but no more often than every 3 days), until the patient reaches target fasting SMPG” |
| Other concomitant intervention | Background therapy: OADs and GLP-1 receptor agonist (if taken) will be considered as NIMP(s). Rescue medication will be considered as NIMP(s): anti-diabetic medication (should be based on Investigator’s decision and local labeling documents). |

| Philis-Tsimikas A (CONCLUDE trial), 2020 | |
| --- | --- |
| Intervention | Experimental:  Insulin degludec (Tresiba®), 200 units/mL, 3 mL prefilled PDS290 (FlexTouch®) pen for subcutaneous injection once daily  Active Comparator:  Insulin glargine (Toujeo®), 300 units/mL, 1.5 mL prefilled Solostar® pen for subcutaneous injection once daily  Background therapy: previous treatment with basal insulin OD or twice daily (BID) ± OADs excluding sulfonylureas/glinides  For patients randomised to IDeg 200 units/mL OD, the daily basal insulin dose should be reduced by 20% from pre-trial dose.  Patients randomised to IGla-300 units/mL should switch unit-to-unit, if they prior to randomization received basal insulin once daily.  For patients that prior to randomization received a BID basal insulin regimen, the following applies:  US patients that prior to randomization received a BID basal regimen with NPH insulin should have their total daily insulin dose reduced by 20% and injected once daily  US patients that prior to randomization received a BID basal regimen with other basal insulin types than NPH should have a unit to unit conversion of their total daily basal insulin dose and injected once daily  EU patients that prior to randomization received a BID basal regimen with any basal insulin type should have their total daily basal insulin dose reduced by 20% and injected once daily |
| Titration period | 16 weeks |
| Strength of insulin | Insulin degludec 200 units/mL, Insulin glargine 300 units/mL |
| Concomitant medications | Oral Anti diabetics Drugs (OADs) excluding sulfonylureas/glinides. |
| Glycemic targets | A target fasting SMPG of 4.0–5.0 mmol/L (71–90 mg/dL). |
| Interval of blood glucose  measurement | Daily pre-breakfast SMPG |
| Calibration of blood  glucose measurement  device | The BG meters use test strips were calibrated to plasma values. Therefore, all measurements performed with capillary blood are automatically calibrated to plasma equivalent glucose values. Only the BG meter provided by Novo Nordisk were used for the measurements required in the protocol. |
| Adjusting insulin doses | Dose adjustment were based on the mean of three fasting SMPG values measured on two days prior to titration and on the day of the visit.  Dose adjustment algorithm   \| Mean of fasting SMPG (mg/dL) from the last 3 measurements \| Insulin dose change \| \| --- \| --- \| \| >162 mg/dl (9 mmol/L) \| + 8 U \| \| 145 – 162 mg/dl (8.1-9 mmol/L) \| + 6 U \| \| 127 – 144 mg/dl (7.1-8 mmol/L) \| + 4 U \| \| 91 – 126 mg/dl (5.1-7 mmol/L) \| + 2 U \| \| 71 – 90 mg/dl (4-5 mmol/L) \| No change \| \| Lowest pre-breakfast SMPG \| Insulin dose change \| \| 56 -70 mg/dl (3.1 – 3.9 mmol/L) \| - 2 U \| \| < 56 mg/dl (<3.1 mmol/L) \| - 4 U \| |
| Interval for insulin adjustments | the insulin dose was adjusted once weekly by the investigator in connection with the scheduled visits/phone contacts. |
| Other concomitant intervention | Oral Anti-diabetic Drugs (OADs): Metformin, Dipeptidyl peptidase-4 inhibitor, Sodium-glucose co-transporter 2 inhibitor, Alpha-glucosidase-inhibitors (acarbose), Thiazolidinediones or marketed oral combination products (not sulfonylureas/glinides) |

| Miura H, 2018 (Kobe Best Basal Insulin Study 2) | |
| --- | --- |
| Intervention | Experimental:  Tresiba ®, FlexTouch (insulin degludec); injection; once daily at the same time every day, up to Week 4 weeks.  Dose titration to achieve target plasma glucose level before breakfast, lunch, dinner, and at bedtime of < 130 mg/dL initially, and subsequently reduced to <110 mg/dL for individuals capable of achieving a reduction based on patients-centered approach.  Background therapy: pre-prandial bolus insulin preparation (change in type not allowed during the trial)  Active Comparator:  Lantus XR, Solostar (Insulin glargine 300 units/mL) up to Week 4 weeks on top of bolus insulin preparation  Dose titration to achieve target plasma glucose level before breakfast, lunch, dinner, and at bedtime of < 130 mg/dL initially, and subsequently reduced to <110 mg/dL for individuals capable of achieving a reduction based on patients-centered approach.  Background therapy: pre-prandial bolus insulin preparation (change in type not allowed during the trial) |
| Titration period | - |
| Strength of insulin | Not described |
| Concomitant medications | Background therapy: pre-prandial bolus insulin preparation (change in type not allowed during the trial) |
| Glycemic targets | Target plasma glucose level before breakfast, lunch, dinner, and at bedtime of < 130 mg/dL initially, and subsequently reduced to <110 mg/dL for individuals capable of achieving a reduction based on  patients-centered approach |
| Interval of blood glucose  measurement | Not described |
| Calibration of blood  glucose measurement  device | Not described |
| Adjusting insulin doses | To avoid unexpected hypoglycemia, new basal insulin dose  Was reduced by 10% compared with that of the previous basal insulin when switching the two formulations.  If the fasting blood glucose level was ≥130 mg/dL (7.2 mmol/L) for more than 3 consecutive days: basal insulin dose was increased by 1 U.  If the fasting blood glucose level was < 70 mg/dL (3.9 mmol/L) for at least 1 day: basal insulin dose was reduced by 2 U. Attending physicians were available to advise patients regarding basal insulin dose |
| Interval for insulin adjustments | Every 3 days for dose increment (if fasting blood glucose ≥130 mg/dL (7.2 mmol/L) and at least 1 day for dose reduction if fasting blood glucose level was < 70 mg/dL |
| Other concomitant intervention | Background therapy: bolus insulin (type not described) |

| Battelino T (InRange trial), 2022 | |
| --- | --- |
| Intervention | Experimental:  Toujeo (Insulin Glargine, 300 units per milliliter [U/ml]) subcutaneous (SC) injection, once daily in the morning before breakfast for 12 weeks on top of rapid acting insulin analog.  Drug: Background therapy: Rapid acting insulin analogs    Active Comparator:  Tresiba (Insulin Degludec, 100 U/ml) SC injection, once daily in the morning before breakfast for 12 weeks on top of rapid acting insulin analog.  Drug: Background therapy: Rapid acting insulin analogs |
| Screening period | 2 weeks |
| Run-in period | 7 weeks |
| Treatment period | 12 weeks (Titration period week 0 to 8 ) , (GCM week 9 to 12 ) |
| Strength of insulin | 100 units/mL degludec, 300 units/mL glargine |
| Concomitant medications | Participants continued their short-acting mealtime insulin analogue (i.e., rapid insulin analogs) which they had used for at least 30 days before the screening visit and continued the same throughout the study. |
| Glycemic targets | Percent (%) time in glucose range of ≥70 to ≤180 mg/dL (≥3.9 to ≤10 mmol/L) at Week 12, obtained using continuous glucose monitoring (CGM) |
| Interval of blood glucose  measurement | The Continuous Glucose Monitoring (CGM) system combined frequent interstitial glucose measurements (every 5 minutes) with ability to analyze glucose levels in real time. |
| Calibration of blood  glucose measurement  device | Participants self-monitor plasma glucose levels  from week – 4 using provided Roche Accucheck® glucometers (Roche Diabetes Care, Inc.,  Mannheim, Germany) and corresponding  accessories (lancet, control solutions and test  strips).” |
| Adjusting insulin doses | During the titration period, doses of Gla-300 or IDeg-100 were titrated until participants achieved the target fasting SMPG of 70 mg/dl or higher to less than 100 mg/dl (≥ 3.9 to < 5.6 mmol/L), while avoiding hypoglycemia.  Dose adjustments for Gla-300 and IDeg-100 were based on median fasting SMPG values from the previous 3 days, including values recorded on the day of titration, as measured by the participants using glucometers. |
| Interval for insulin adjustments | At least weekly (but no more often than every 3 days) until participants achieve the target fasting SMPG of C 70 to\100 mg/dL (C 3.9 to\5.6 mmol/L) while avoiding hypoglycemia episodes |
| Other concomitant intervention | Participants continued their short-acting mealtime insulin analogue (i.e., rapid insulin analogs) which they had used for at least 30 days before the screening visit and continued the same throughout the study |

#
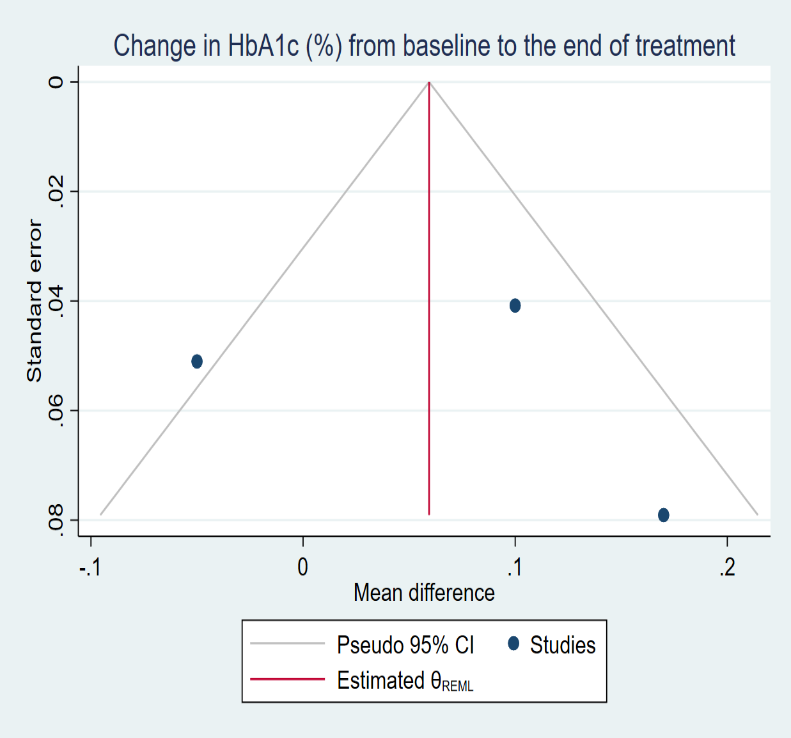
Appendix 5: Assessment of publication bias

Figure 4A Funnel plot for change in HbA1c


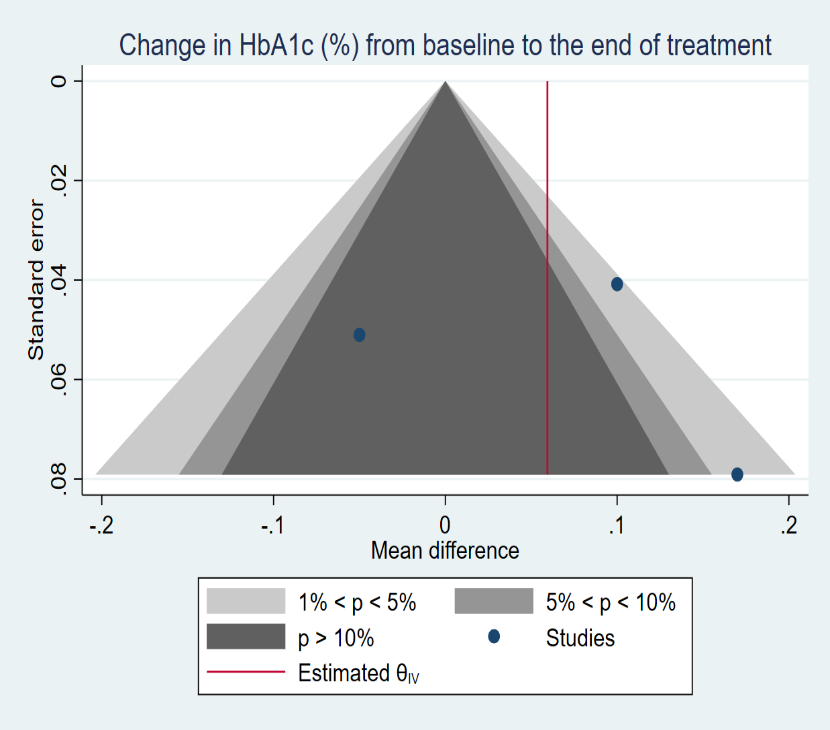


 Figure 4B: Contour-enhanced funnel plot for change in HbA1c

Figure 4C: Funnel plot for change in fasting plasma glucose (FPG
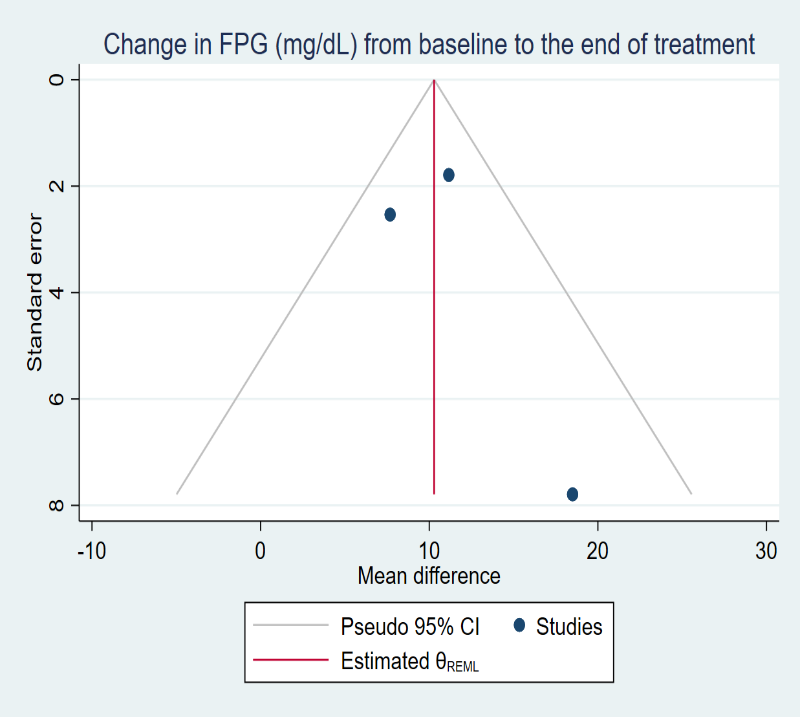
)


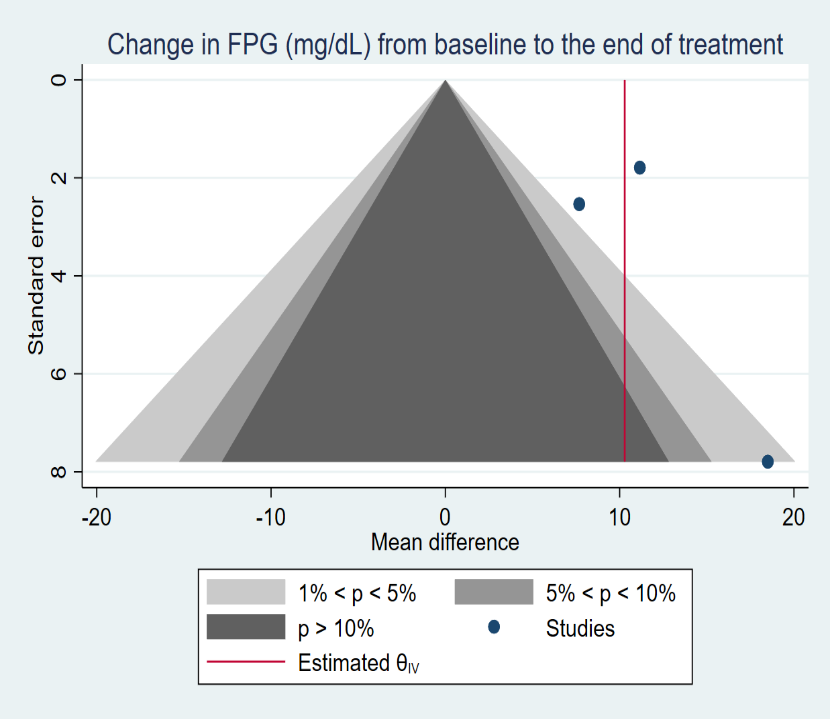


Figure 4D: Contour-enhanced for change in FPG


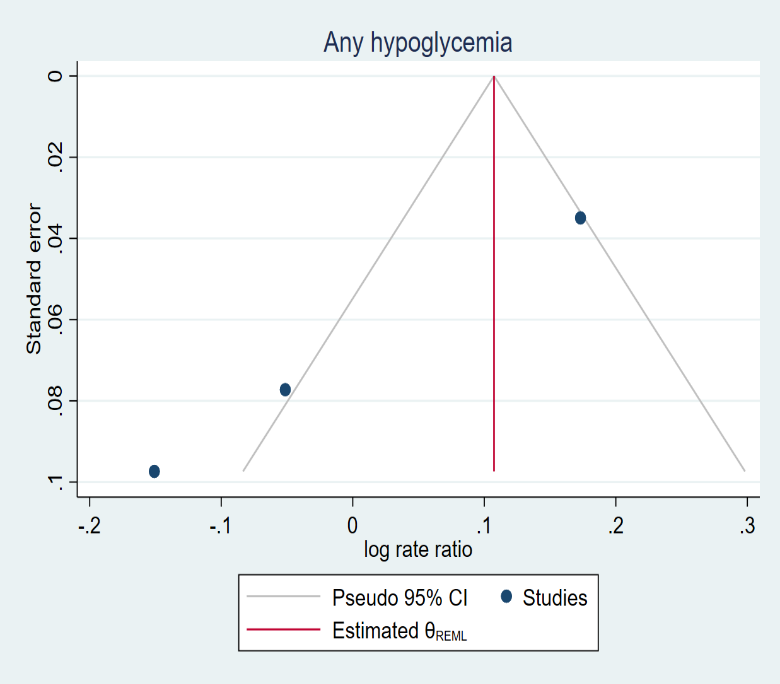
Figure 4E: Funnel plot for any time hypoglycemia


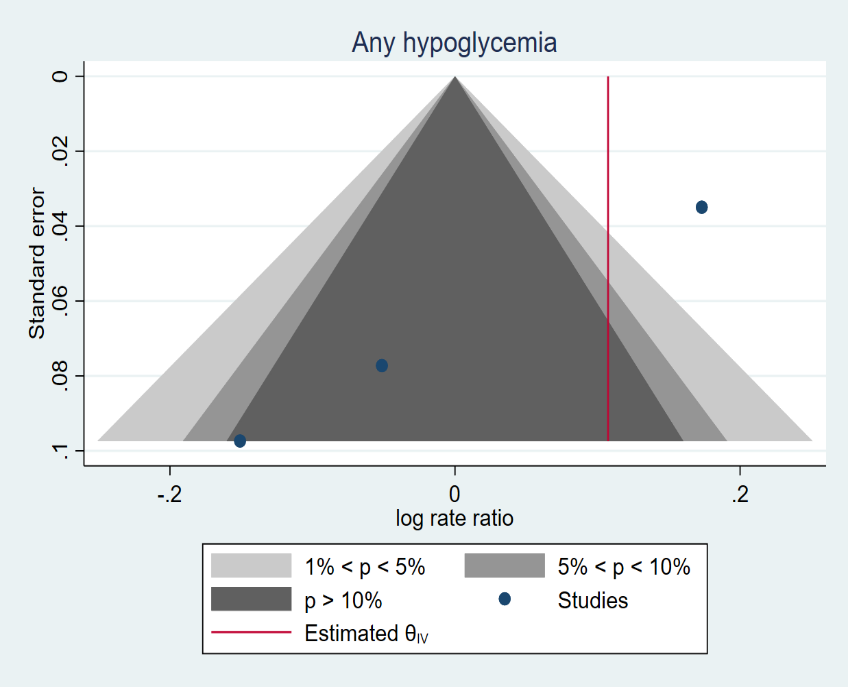


Figure 4F: Contour-enhanced for any time hypoglycemia


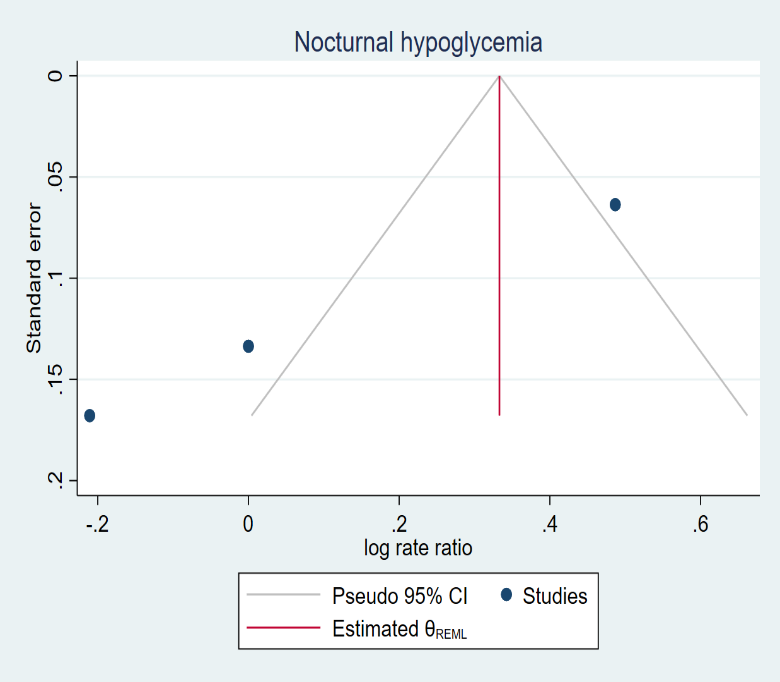
Figure 4G: Funnel plot for nocturnal hypoglycemia


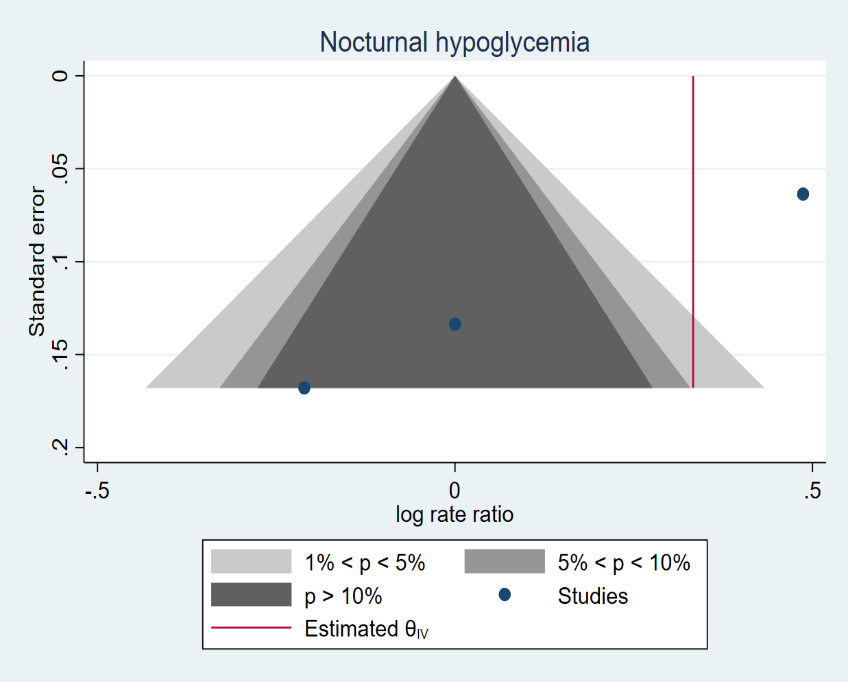


Figure 4H: Contour-enhanced for nocturnal hypoglycemia


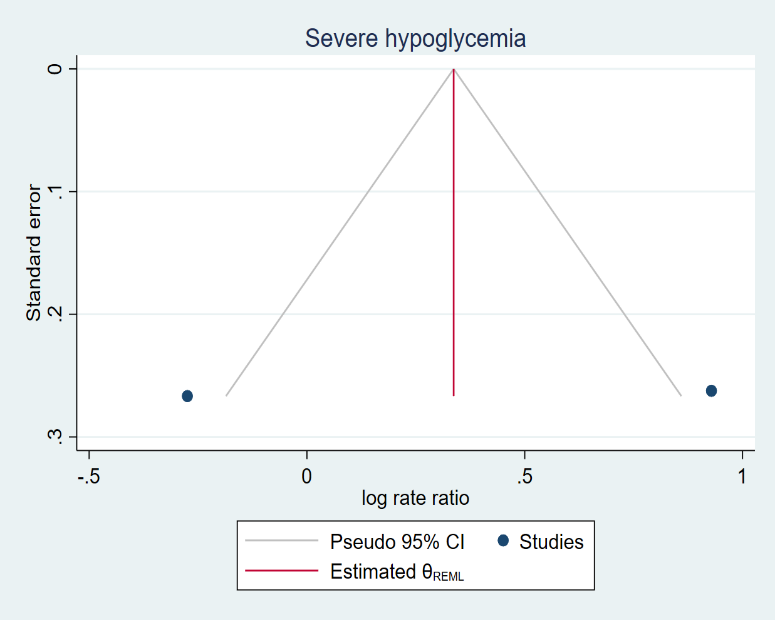
 Figure 4I: Funnel plot for severe hypoglycemia


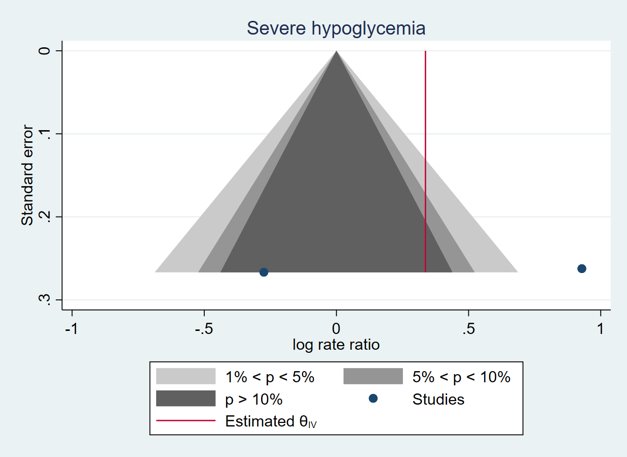


Figure 4J: Contour-enhanced plot for severe hypoglycemia

# Appendix 6: Investigation of heterogeneity and outliers


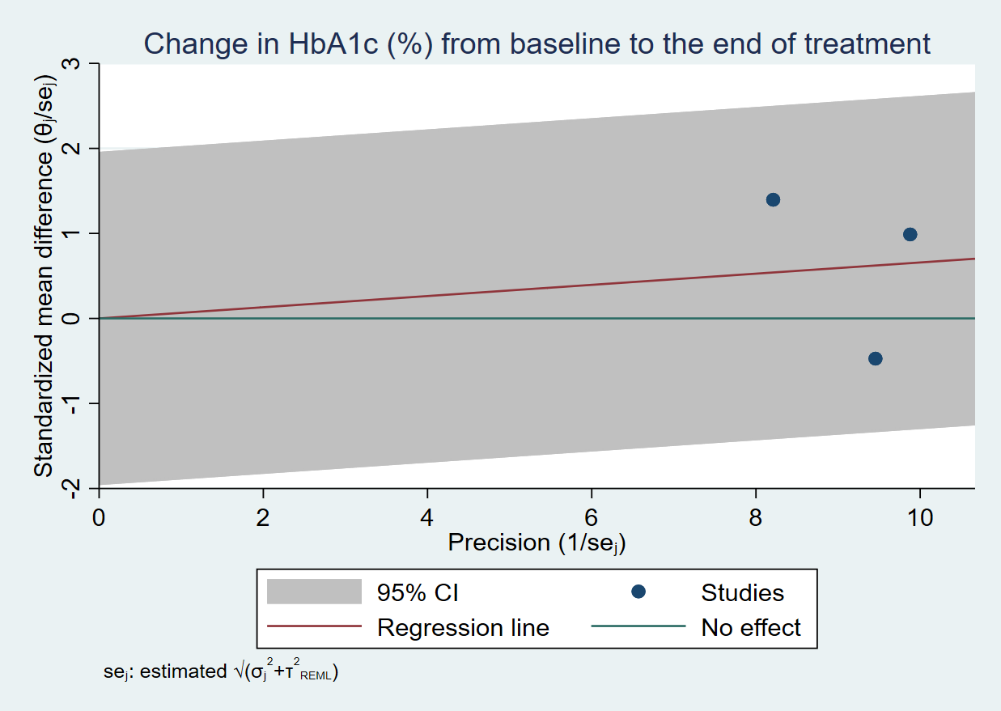


Figure 5A: Galbraith plot for change in HbA1c


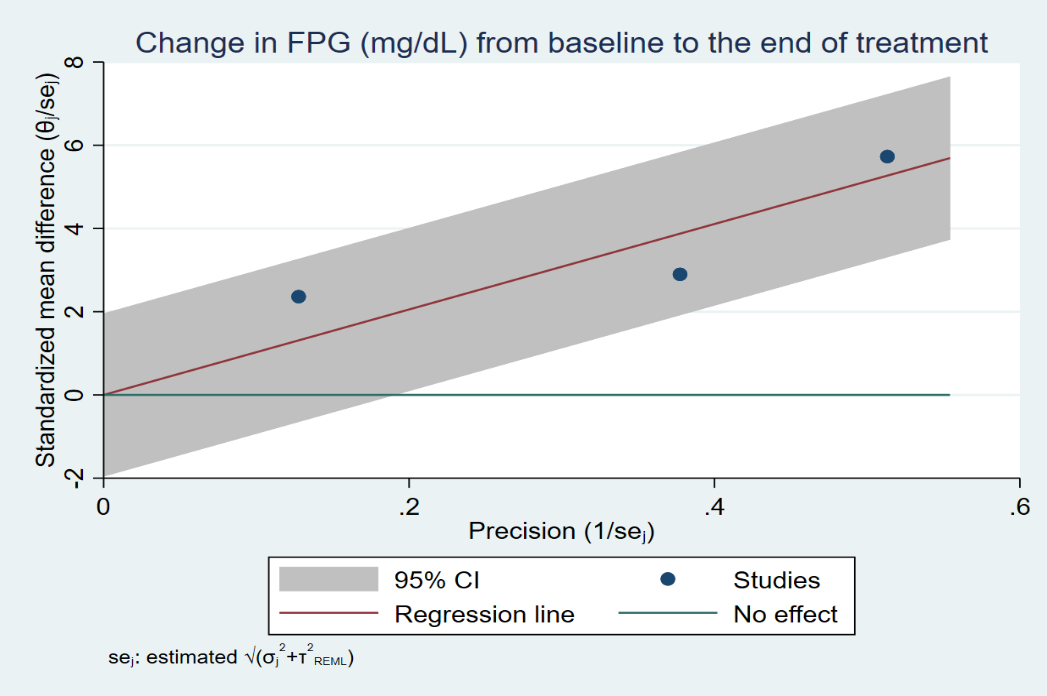


  Figure 5B: Galbraith plot for change in FPG


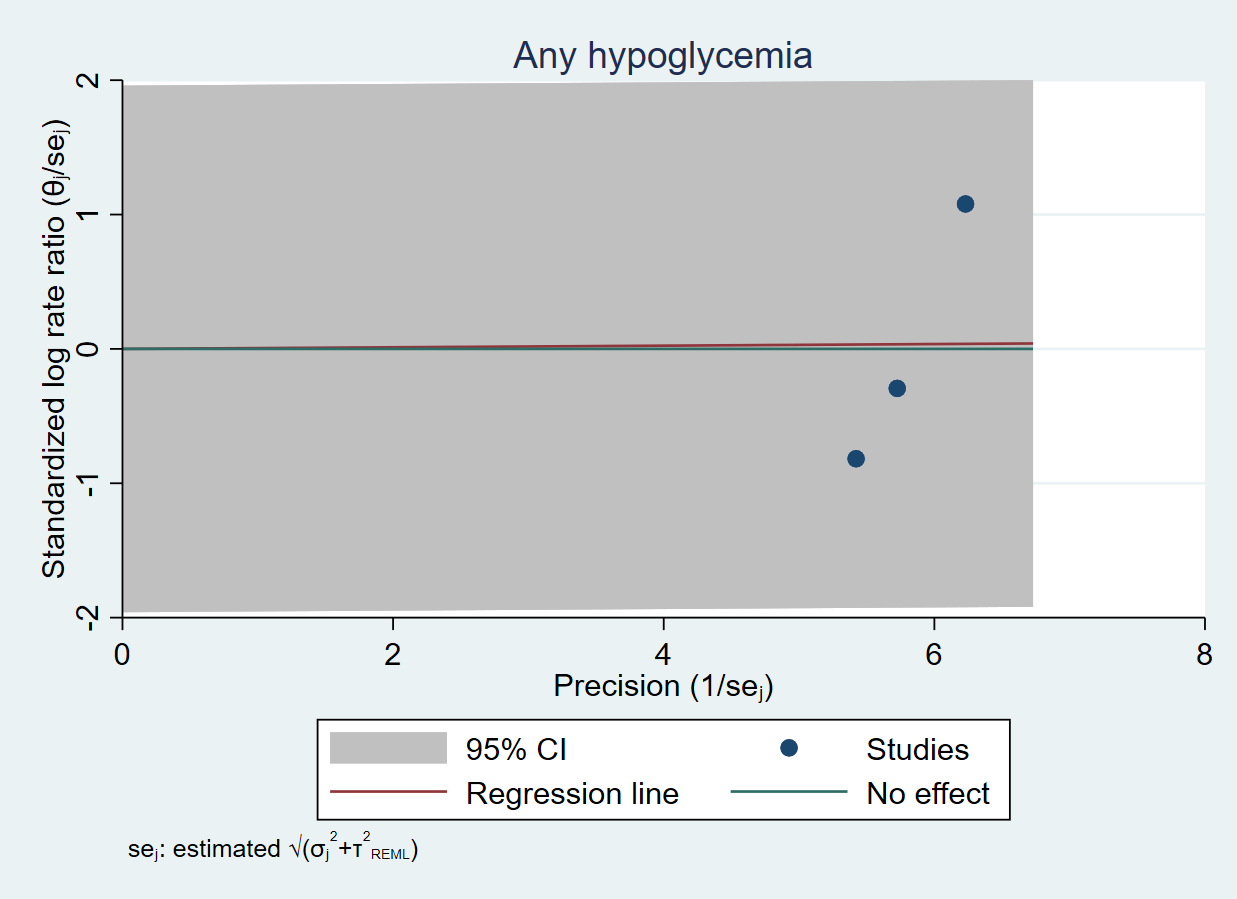
 Figure 5C: Galbraith plots for any hypoglycemia


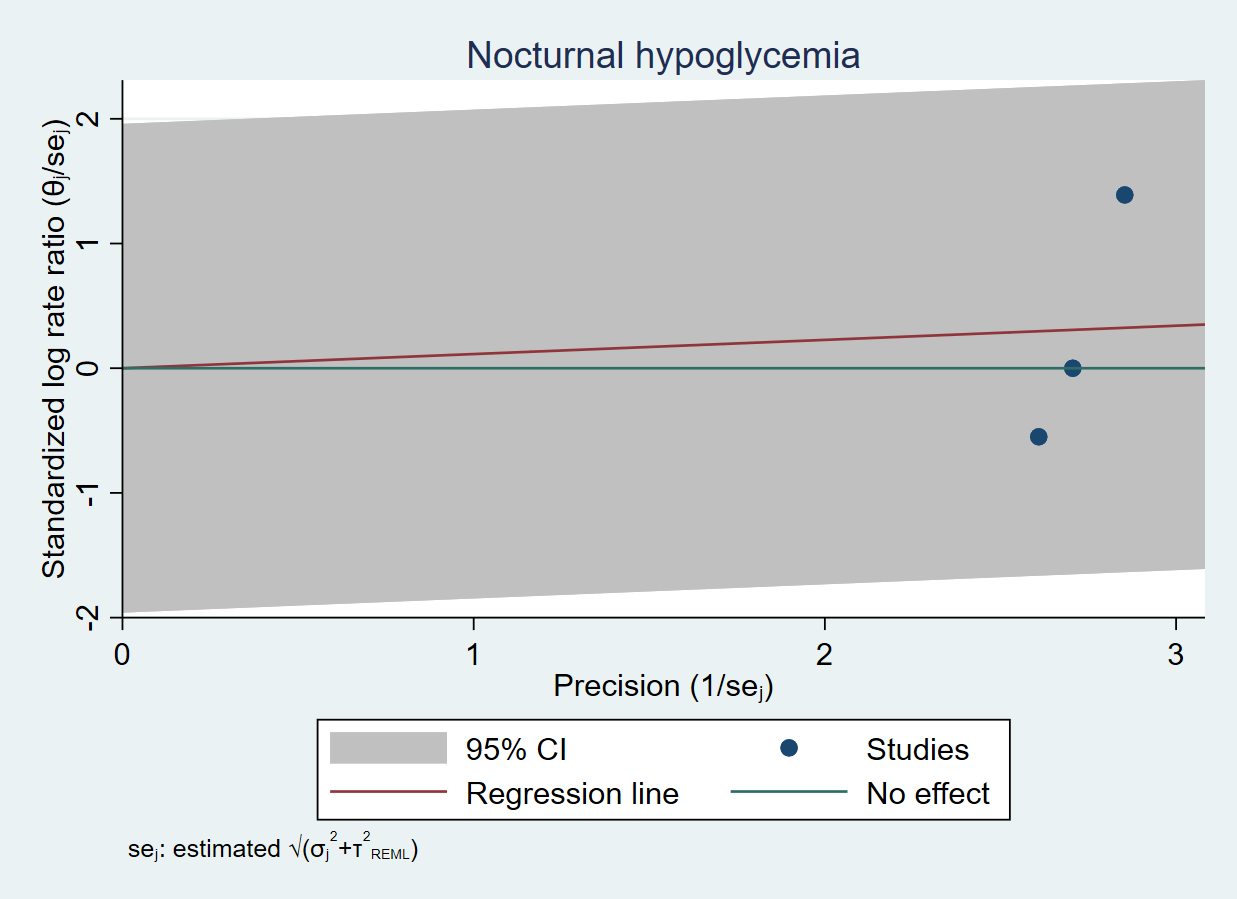


Figure 5D: Galbraith plots for nocturnal hypoglycemia


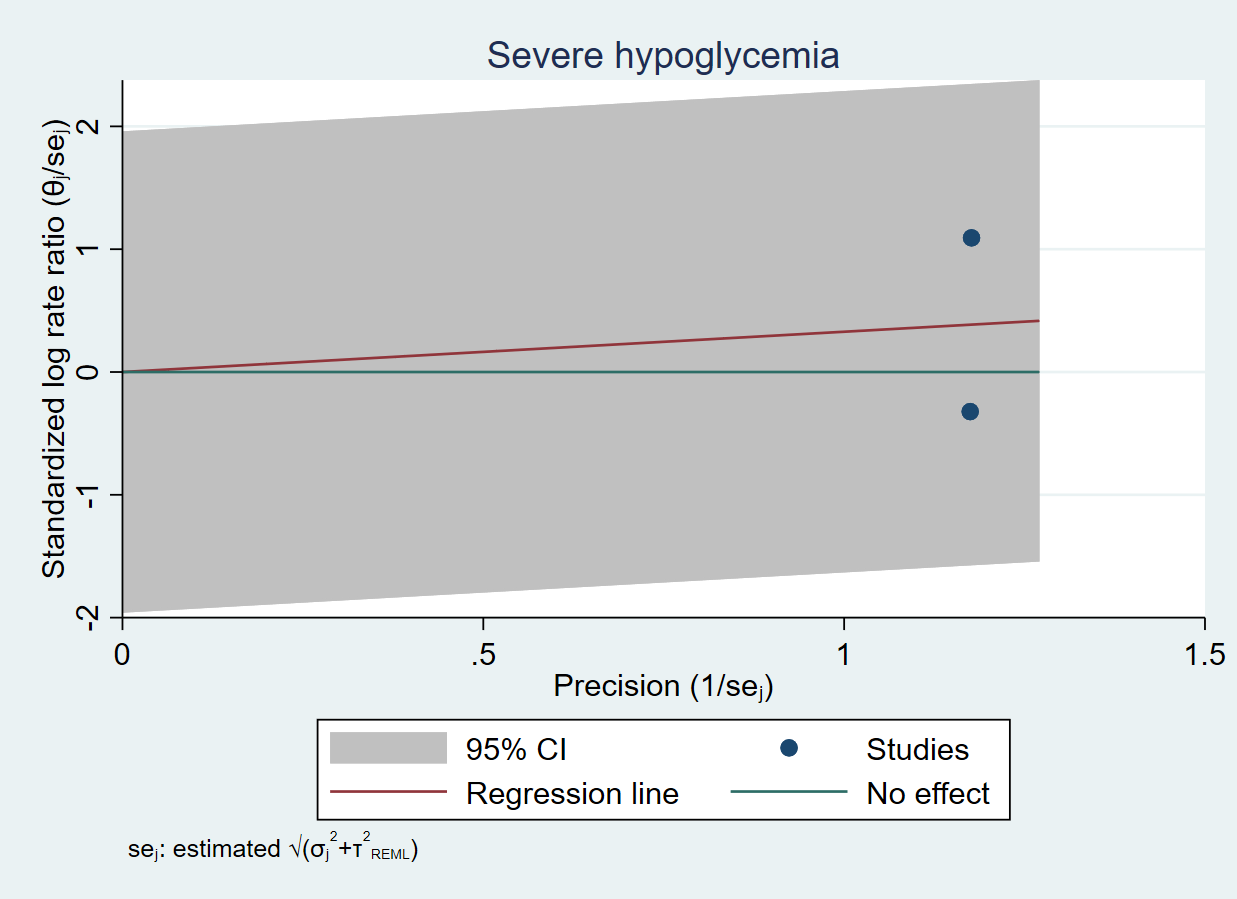
 Figure 5E: Galbraith plots for severe hypoglycemia

#
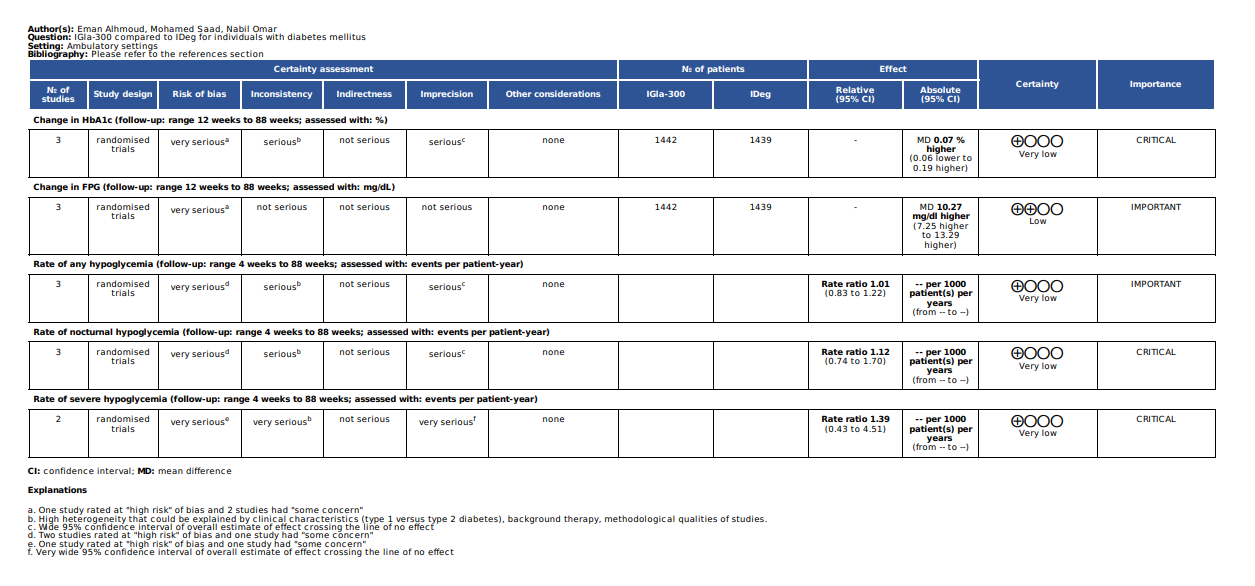
Appendix 7: GRADE evidence profile
